# Acoustofluidic Synthesis of Particulate Nanomaterials

**DOI:** 10.1002/advs.201900913

**Published:** 2019-08-27

**Authors:** Po‐Hsun Huang, Shuaiguo Zhao, Hunter Bachman, Nitesh Nama, Zhishang Li, Chuyi Chen, Shujie Yang, Mengxi Wu, Steven Peiran Zhang, Tony Jun Huang

**Affiliations:** ^1^ Department of Mechanical Engineering and Materials Science Duke University Durham NC 27708 USA; ^2^ Department of Engineering Science and Mechanics Pennsylvania State University University Park PA 16802 USA

**Keywords:** acoustic streaming, acoustofluidics, nanomaterials, nanoparticles, synthesis

## Abstract

Synthesis of nanoparticles and particulate nanomaterials with tailored properties is a central step toward many applications ranging from energy conversion and imaging/display to biosensing and nanomedicine. While existing microfluidics‐based synthesis methods offer precise control over the synthesis process, most of them rely on passive, partial mixing of reagents, which limits their applicability and potentially, adversely alter the properties of synthesized products. Here, an acoustofluidic (i.e., the fusion of acoustic and microfluidics) synthesis platform is reported to synthesize nanoparticles and nanomaterials in a controllable, reproducible manner through acoustic‐streaming‐based active mixing of reagents. The acoustofluidic strategy allows for the dynamic control of the reaction conditions simply by adjusting the strength of the acoustic streaming. With this platform, the synthesis of versatile nanoparticles/nanomaterials is demonstrated including the synthesis of polymeric nanoparticles, chitosan nanoparticles, organic–inorganic hybrid nanomaterials, metal–organic framework biocomposites, and lipid‐DNA complexes. The acoustofluidic synthesis platform, when incorporated with varying flow rates, compositions, or concentrations of reagents, will lend itself unprecedented flexibility in establishing various reaction conditions and thus enable the synthesis of versatile nanoparticles and nanomaterials with prescribed properties.

## 1 Introduction

Nanoparticles (NPs) and particulate nanomaterials (NMs) hold tremendous promise for numerous applications including energy, imaging, and nanomedicine.^1^, ^2^, ^3^, ^4^, ^5^, ^6^, ^7^ For example, polymeric NPs are widely utilized in vivo to achieve targeted drug release,^8^ while quantum dots improve resolution and brightness for bioimaging/detection.^9^, ^10^ In order for these applications to be realized, NPs/NMs must be synthesized in a controllable manner to tailor desired physicochemical properties (e.g., size, shape, charge, stability, polydispersity, encapsulation efficiency, chemical composition, crystal structure, etc.).^11^, ^12^, ^13^, ^14^ Conventionally, NPs/NMs are synthesized through bulk synthesis methods in the form of stirring, shaking, pipetting, or vortexing to actively mix reagents together. These bulk methods, however, lack fine control over reaction conditions, thus leading to undesirable quality and batch‐to‐batch reproducibility. Therefore, synthesis methods capable of precisely dictating reaction conditions are critical to reproducibly yielding NPs/NMs with tailored physicochemical properties.

Owing to their ability to precisely handle minute amounts of reagents, microfluidics‐based methods provide powerful solutions to the issues associated with bulk synthesis.^15^, ^16^, ^17^, ^18^, ^19^, ^20^ Among the most widely used microfluidics‐based synthesis methods are hydrodynamic focusing^21^ and droplet‐based methods.^22^ Relying on passive, diffusion‐controlled mixing between two reagents, hydrodynamic‐focusing‐based methods can synthesize NPs/NMs with controlled properties;^23^, ^24^, ^25^, ^26^, ^27^ however, due to the boundary‐based nature of diffusive mixing, they remain difficult to perform syntheses involving the mixing of multiple reagents, limiting the variety of NPs/NMs they can synthesize. Droplet‐based methods (particularly water‐in‐oil systems), which mix reagents based on chaotic advection and recirculation within nano/picoliter droplets,^28^, ^29^, ^30^, ^31^, ^32^ can accomplish syntheses that require the mixing of multiple reagents, but they need to stabilize droplets^33^ with surfactants, which may unfavorably react with the reagents and therefore compromise the quality of synthesized NPs/NMs.^34^, ^35^ Additionally, downstream phase separation is first needed to isolate the water phase (i.e., droplets containing NPs/NMs) from the oil phase upon the completion of synthesis, which further complicates the design and operation of droplet‐based methods.^36^, ^37^ In addition to hydrodynamic focusing and droplet‐based methods, other microfluidics‐based^38^, ^39^, ^40^ and millifluidics‐based^41^, ^42^, ^43^, ^44^ synthesis methods have also shown their capabilities for NPs/NMs synthesis, but most of them control their reaction conditions (i.e., the mixing time) by altering the flow rate ratio of reagents. As a result, the independent influence of either the mixing time, or the bulk volume ratio of reagents on the synthesized NPs/NMs cannot be uncovered. Moreover, most of the existing synthesis methods rely on passive, partial mixing of reagents; this partial mixing may yield significant amounts of unreacted reagents. These unused reagents could lead to undesired downstream reactions, which adversely alter the properties of NPs/NMs synthesized, thus necessitating an immediate postreaction purification. Hence, one can envision a synthesis platform—based on an active, complete mixing strategy—that can reproducibly synthesize NPs/NMs with uniform, yet controllable properties, while maintaining flexibility in dictating reaction conditions.

Here, we present an NP/NM synthesis platform based on an acoustofluidic (i.e., the fusion of acoustics and microfluidics)^45^, ^46^, ^47^, ^48^, ^49^, ^50^, ^51^, ^52^, ^53^, ^54^, ^55^, ^56^ strategy for rapid, adaptable, and complete mixing of reagents. Our acoustofluidic device actively blends reagents together utilizing acoustic streaming induced by the oscillation of sharp‐edge structures in the microchannel. The acoustic streaming vigorously agitates fluids and facilitates rapid mass transport, thereby achieving complete mixing of reagents. Using this acoustofluidic synthesis method, we demonstrate the syntheses of various NPs/NMs including polymeric NPs, chitosan NPs, nonspherical hybrid NMs, metal–organic frameworks (MOFs) biocomposites, and lipid/DNA complexes. Not only can our acoustofluidic platform minimize the influence of downstream reactions from residual reagents, but it can also adapt the mixing time and efficiency without changing the flow rates of reagents. It can also keep the mixing time constant while changing the flow rate ratio of reagents. Moreover, it can mix several reagents at once or in a prescribed order at preferred flow rate ratios, which, along with the controllable mixing time and efficiency, will allow one to synthesize versatile NPs/NMs with highly uniform, tailored properties. Our device is simple to fabricate and operate and offers controllable reaction conditions; these features, when combined with the capabilities mentioned above, render our acoustofluidic synthesis platform indispensable for future fabrication of NPs/NMs that are unattainable through existing synthesis methods.

## 2 Results and Discussion

### 2.1 Principle of Acoustofluidic Nanomaterial Synthesis

**Figure 1:**
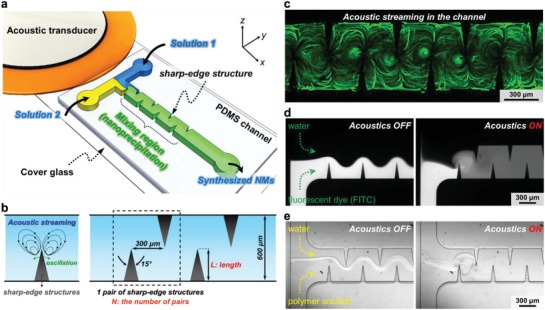
Design and concept of the acoustofluidic platform for nanoparticle (NP) synthesis. a) Schematic showing the acoustofluidic synthesis device. The device is composed of a PDMS microfluidic channel constructed with multiple pairs of sharp‐edge structures, a glass slide, and an acoustic transducer. When flowing through the channel, Solution 1 and Solution 2 are rapidly yet completely mixed in the presence of the acoustic streaming effect, thus resulting in the self‐assembly of PLGA‐PEG NPs. b) Schematic showing the generation of acoustic streaming and design of sharp‐edge structure. c) Stacked fluorescent image displaying the acoustic streaming generated in the acoustofluidic device. d) Fluorescent images showing the mixing of two fluids. When the acoustic transducer is off, an unmixed, laminar flow pattern is observed, whereas a complete mixing of two fluids is achieved when the acoustic transducer is on. Results shown in (c) and (d) are obtained under the same conditions: the driving voltage of 20 V_PP_, the driving frequency of 4.0 kHz, and the flow rate of 10 µL min^−1^ for each stream. e) Bright‐field images showing the mixing of water and PLGA‐PEG precursor solution. Similarly, when the acoustic transducer is off, the two solutions form a laminar flow, where NPs may also be synthesized through a diffusion‐based mixing between the two solutions. Once the transducer in turned on, the two solutions are rapidly mixed without any fluidic interface observed; this rapid and complete mixing leads to an exchange between the solvent and water, also known as solvent exchange, and therefore produces PLGA‐PEG NPs.

We synthesize NPs/NMs by rapidly and completely mixing two reagents using the acoustic streaming effect generated in our acoustofluidic device (**Figure** **1**a). The acoustofluidic device consists of a single‐layer polydimethylsiloxane (PDMS) channel with several pairs of sharp‐edge structures protruding from its sidewalls, a cover glass, and an acoustic transducer. Electrically actuating the acoustic transducer causes the sharp‐edge structures to oscillate and generate a pair of counter‐rotating vortices, also known as acoustic streaming, around their tips (Figure 1b,c).^57^, ^58^, ^59^, ^60^, ^61^, ^62^, ^63^, ^64^ The acoustic streaming breaks the interface between laminar fluids and facilitates mass transport across the channel width, allowing for a rapid, uniform mixing of reagents and achieving NPs/NMs synthesis. The mixing of two solutions using our device is first visualized by coflowing fluorescent dye (FITC‐dextran) and deionized (DI) water into the channel (Figure 1d). When the transducer is off (i.e., in the absence of acoustic streaming), the two solutions form a side‐by‐side laminar flow; upon switching the transducer on (i.e., in the presence of acoustic streaming), they are rapidly and completely blended together after passing through the first pair of sharp‐edge structures (Figure 1d), with a mixing time of ≈80 ms (see Note S1 in the Supporting Information for the estimation of mixing time). To validate our concept for NPs/NMs synthesis, we first synthesize polymeric NPs by mixing water with a precursor of poly(lactide‐*co*‐glycolide)‐block‐poly(ethylene glycol)‐carboxylic acid (PLGA‐PEG‐COOH). Likewise, when the transducer is off, the water and polymer solution form a laminar flow; once the transducer is switched on, the resultant acoustic streaming breaks the interface of the water and polymer solution and rapidly mixes them together (Figure 1e). This rapid mixing of water and polymer precursor leads to the self‐assembly of NPs due to flash nanoprecipitation.^65^

Since our acoustofluidic platform synthesizes NPs/NMs based on active, complete mixing as opposed to passive, partial mixing of reagents, its mixing performance predominately affects the size and uniformity of NPs/NMs. The mixing performance is directly related to the strength of the acoustic streaming generated; stronger acoustic streaming dramatically improves mixing performance, which, in turn, yields NPs/NMs with smaller size and better uniformity than weaker acoustic streaming does. Therefore, we seek to optimize the mixing performance of our platform by investigating the size dependence of PLGA‐PEG NPs on its operational and design parameters, including the driving frequency of the transducer and the length and number of sharp‐edge structures (Figure 1b). In these optimization experiments, the 10 mg mL^−1^ solution of PLGA_10K_‐PEG_5K_ and water are infused into the device at the flow rates of 1 and 10 µL min^−1^, respectively. Through these parametric studies, we identify 4.0 kHz and 300 µm as the optimal driving frequency and optimal length of sharp‐edge structures, respectively, since these parameters, when adopted, generate the smallest and most uniform PLGA‐PEG NPs, among those tested (Note S2 and Figures S1 and S2, Supporting Information).

**Figure 2:**
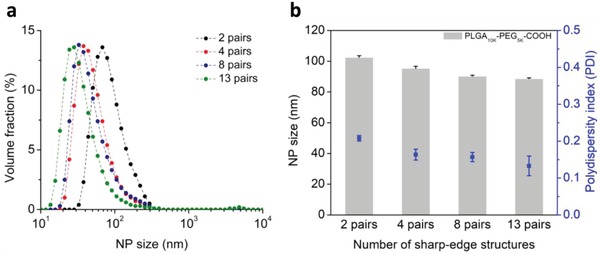
Determination of an optimal number of sharp‐edge structures. a) Size distribution and b) average size for PLGA‐PEG NPs produced using acoustofluidic devices with different numbers of sharp‐edge structures. As the number of sharp‐edge structures increases from 2 to 13 pairs, the size distribution shifts toward a smaller size and becomes tighter. These results demonstrate that as the number of sharp‐edge structures increases from 2 to 13 pairs, the average size is reduced modestly from 103 to 89 nm while the size uniformity (polydispersity index) is improved noticeably from 0.21 to 0.13, suggesting an optimal number of 13 pairs of sharp‐edge structures. Experiments for these results are carried out under the following conditions: the driving voltage of 20 V_PP_, and the flow rate of 1 and 10 µL min^−1^, respectively, for the water and polymer solution. Error bars denote standard deviation from at least three experiments (*n* ≥ 3).

We believe that the number of sharp‐edge structures may also play a critical role in the mixing performance. Each sharp‐edge structure, when acoustically oscillated, induces acoustic streaming and, as such, serves as a miniature vortex mixer. Therefore, if there are four pairs of sharp‐edge structures in the channel (as depicted in Figure 1a), then the two solutions are essentially mixed by eight microscale vortex mixers as they flow through the channel. Conversely, increasing the number of sharp‐edge structures can also increase the fluidic length (the path the two solutions flow through), thus increasing the diffusion length (i.e., increase the diffusion time). This increased diffusion time may influence the mixing performance, but only when the acoustic field is off; when the acoustic field is off, the mass transport between two solutions relies entirely on the diffusion occurring at the interface of the two solutions. Once the acoustic field is switched on, the diffusion length and time are considered negligible compared to the dominant acoustic streaming, which breaks the interface and violently agitate the fluid;^57^, ^66^ the mixing performance in the acoustofluidic setup is thus determined primarily by the strength of the acoustic streaming and the number of sharp‐edge structures. Besides, this work is devoted to utilizing the acoustofluidic mixing as opposed to the diffusion‐based mixing to synthesize NPs/NMs and therefore, we seek to identify the optimal number of sharp‐edge structures based on the synthesis performance with the acoustofluidic mixing on. On this basis, we thus hypothesize that, by increasing the number of sharp‐edge structures inside the channel, the mixing performance, particularly the mixing uniformity, could be substantially improved. To confirm this hypothesis, we synthesize PLGA‐PEG NPs using devices with varying numbers of sharp‐edge structures. Dynamic light scattering (DLS) analysis indicates that as the number of sharp‐edge structures increases from 2 to 13 pairs, the NP size decreases from 102.8 ± 1.2 to 88.61 ± 0.6 nm with the polydispersity index (PDI) improving from 0.21 ± 0.01 to 0.13 ± 0.02, respectively (**Figure** **2**). Increasing the number of sharp‐edge structures modestly reduces the NP size while markedly improving the size uniformity, thus confirming the hypothesis as well as identifying 13 pairs as the optimal number of sharp‐edge structures for the present device design. Moreover, accommodating more sharp‐edge structures in the channel may also ensure complete mixing of fluids at higher flow rates, which may not be possible if there are fewer sharp‐edge structures (e.g., eight of them). Based on this result, acoustofluidic devices with 13 pairs of 300 µm long sharp‐edge structures are employed and activated at the frequency of 4.0 kHz in all subsequent synthesis experiments, unless otherwise noted. Detailed experimental setup, materials (including polymers and chemical reagents), and characterization methods for synthesized NPs/NMs are provided in the Experimental Section.

### 2.2 Synthesis of PLGA‐PEG NPs by Acoustofluidic Device

**Figure 3:**
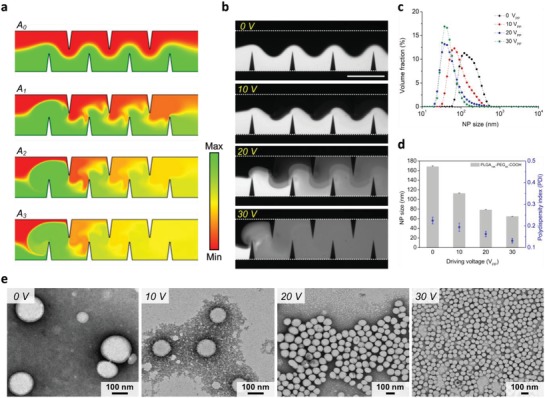
Effect of the mixing performance of the acoustofluidic device on the size of PLGA‐PEG NPs. a) Simulation results and b) experimental results showing the concentration distribution, i.e., the mixing performance, under different driving voltages. The experimental results are qualitatively consistent with the simulation results and suggest that the mixing performance of our acoustofluidic device can be adjusted by controlling the driving voltage. c) Size distribution, d) average size, and e) TEM images of NPs synthesized by our acoustofluidic device under different driving voltages. As the driving voltage increases, the size distribution becomes narrower and the NP size is reduced markedly. Additionally, the volume fraction of NPs also rises as the driving voltage increases. These results demonstrate that the NP size can be controlled by adjusting the driving voltage, i.e., the mixing performance. Experiments are conducted using 10 mg mL^−1^ PLGA_10K_‐PEG_5K_ under the driving frequency of 4.0 kHz and the flow rate of 10 µL min^−1^ for both water and PLGA‐PEG solution. Error bars denote standard deviation from at least three experiments (*n* ≥ 3).

For our acoustofluidic synthesis device, the strength of acoustic streaming can be adjusted by changing the driving voltage of the transducer, thereby altering the mixing performance^59^, ^66^, ^67^ and the physicochemical properties of synthesized NPs. To demonstrate the control over the quality of NPs by altering the mixing performance, we synthesize PLGA‐PEG NPs with our acoustofluidic device under different driving voltages. With the numerical model we previously developed,^58^, ^59^ we first predict the concentration distribution of solutions (i.e., the mixing performance) under different vibration amplitudes of the sharp‐edge structures (**Figure** **3**a); as the vibration amplitude is increased from A_0_ to A_3_, enhanced acoustic streaming improves the mixing performance. Improved mixing performance due to increased driving voltages is also confirmed in experiments (Figure 3b), where complete mixing is achieved after the first pair of sharp‐edge structures when the device is activated with 30 V_PP_; this observation also suggests that, in terms of improving the mixing performance, increasing the driving voltage applied to the transducer may be more efficient than increasing the number of sharp‐edge structures. Nevertheless, as mentioned in the previous section, we must employ more pairs of sharp‐edge structure not only to improve the mixing uniformity but also to guarantee complete mixing of liquids at high flow rates. Additionally, we note that the simulation results deviate slightly from the experimental ones; they both indicate improved mixing performance by increasing the vibration amplitude. The deviation is most likely due to the fact that in the numerical model, we could specify only the degree of vibration, as opposed to an exact vibration amplitude corresponding to a given driving voltage. Dynamic light scattering analysis shows that as the driving voltage increases from 0 to 30 V_PP_, the size distribution narrows and the NP size decreases strikingly from ≈170 nm down to 65 nm (Figure 3c). When the acoustofluidic mixing is off (0 V_PP_), NPs can still be formulated relying solely on the slow, diffusion‐based mixing occurring in the interface of the water and polymer solution, with an average size of 168.3 ± 1.5 nm (polydispersity index = 0.22 ± 0.02). The diffusion‐based mixing achieves complete solvent exchange on a time scale longer than that for polymer aggregation; as such, the polymers nucleate less nanoparticle seeds and tend to aggregate on those seeds, eventually forming larger NPs (see Figure S4 in the Supporting Information).^25^ Activating our device at 10 V_PP_ alone can lead to a significant decrease in the NP size by 33% to 112.7 ± 0.7 nm (polydispersity index = 0.19 ± 0.02). Increasing the driving voltage to 20 V_PP_ further reduces the NP size to 78.4 ± 0.5 nm (polydispersity index = 0.16 ± 0.01). When driven at 30 V_PP_, our device produces PLGA‐PEG NPs as small as 64.7 ± 0.5 nm with an average polydispersity index of 0.13 ± 0.01 (Figure 3d). The reduces in size and polydispersity due to increased driving voltages are expected and can be attributed to the rapid solvent exchange facilitated by enhanced acoustic streaming (Figure S4, Supporting Information). At high driving voltages, the solvent exchange is completed on a time scale shorter than that for polymer aggregation and as a result, the polymers nucleate many more nanoparticle seeds. Additionally, the rapid solvent exchange increases the barrier for the polymers to adsorb onto the nanoparticle seeds and supports a uniform aggregation process,^25^, ^68^ thus preventing NPs from growing too large and promotes the formation of NPs that are small and uniform in size. The polydispersity is thus decreased because of the improved size uniformity. The size and uniformity of the NPs produced under different driving voltages are also confirmed by transmission electron microscopy (TEM) examination (Figure 3e). These results show that our acoustofluidic method can reproducibly yield PLGA‐PEG NPs with a size variation of ±1 nm and a variation of ±0.02 in polydispersity index among independent experiments, when using the same batch of PLGA‐PEG precursor. This degree of reproducibility can also be achieved for samples produced using another batch of precursor (Note S3 and Figure S5, Supporting Information), thus demonstrating the reliability and robustness of our acoustofluidic synthesis platform. We also note that the second batch produces PLGA‐PEG NPs that are overall smaller than those produced using the first batch. In addition to the size and uniformity, the zeta potential (ζ‐potential) for the PLGA‐PEG NPs synthesized is also examined. The ζ‐potential decreases as the driving voltage is increased, that is, the NP size is reduced (Figure S6, Supporting Information). Note, however, that the overall ζ‐potential is relatively low regardless of the driving voltage, because this specific precursor has an intrinsically low ζ‐potential.^69^ Despite the low ζ‐potential, these results demonstrate that by adjusting the driving voltage, our platform can prepare NPs with uniform (low polydispersity index), controllable size in a reproducible fashion (small size variation among different experimental batches).

**Figure 4:**
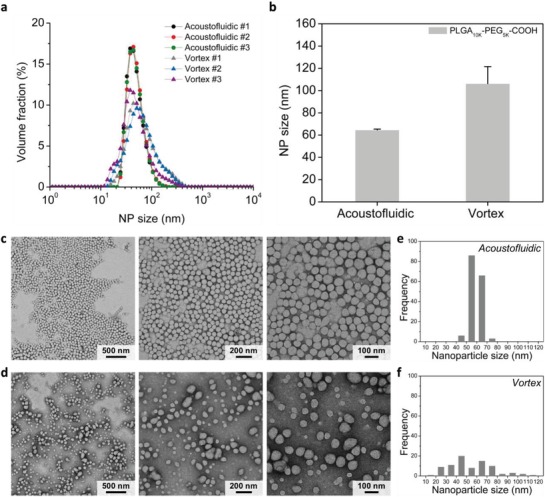
Comparison of PLGA‐PEG NPs synthesized by vortex mixing and acoustofluidic mixing. a) Size distribution and b) average size of PLGA‐PEG NPs synthesized by the two different methods. TEM images of PLGA‐PEG NPs prepared by c) acoustofluidic device and d) vortex mixing. Histograms obtained by analyzing the TEM images with ImageJ showing the size distribution of the PLGA‐PEG NPs synthesized by e) acoustofluidic device and f) vortex mixing. Overall, these results demonstrate that compared to vortex mixing, our acoustofluidic device can reproducibly yield PLGA‐PEG NPs with higher volume fraction, smaller size, and higher uniformity. Acoustofluidic experiments are performed using 10 mg mL^−1^ PLGA_10K_‐PEG_5K_ under the driving frequency of 4.0 kHz, the driving voltage of 30 V_PP_, and the flow rate of 10 µL min^−1^ for both the water and PLGA‐PEG solution. For the vortex mixing, 50 µL PLGA_10K_‐PEG_5K_ and water of the same volume are added into a 1.5 mL centrifuge tube and mixed on a vortex mixer for 1 min. Error bars denote standard deviation from at least three experiments (*n* ≥ 3).

As a comparison to our acoustofluidic device, we synthesize PLGA‐PEG NPs using vortex mixing, one of the conventional bulk synthesis methods; 50 µL of the same PLGA‐PEG solution is mixed with 50 µL water on a vortex mixer for 1 min. The PLGA‐PEG NPs prepared by our acoustofluidic platform feature narrower size distributions than those prepared by vortex mixing, with the size‐distribution curves nearly overlapping (**Figure** **4**a). Our acoustofluidic device produces NPs with an average size of 64.7 ± 0.7 nm, which is ≈39% smaller than those prepared by vortex mixing (106.3 ± 15.2 nm) (Figure 4b). TEM images confirm that our acoustofluidic device generates smaller NPs of uniform size, and that vortex mixing prepares larger NPs with a broader range of sizes (Figure 4c,d). Quantitatively analyzing the TEM images, we further verify that the size of the NPs prepared by the acoustofluidic device falls into a narrow size range with most of the NPs being around 50–70 nm (Figure 4e), while vortex mixing yields NPs with sizes ranging widely from 10 to 120 nm (Figure 4f). It is worth pointing out that the vortex mixing, to some extent, is similar to the acoustofluidic mixing at lower voltages, where the solvent exchange is completed on a time scale longer than that for polymer aggregation; as a result, it produces NPs with larger sizes and wider size distribution, as elucidated in Figure S4 in the Supporting Information. Additionally, we further examine the quality of NPs we synthesized by characterizing the ζ‐potential of NPs prepared by the acoustofluidic device at 30 V_PP_ and vortex mixing, which are −2.7 ± 0.2 and −3.8 ± 1.3 mV, respectively (Figure S6, Supporting Information). This insignificant difference in the ζ‐potential between the two synthesis methods is expected, given the intrinsically low ζ‐potential for this specific polymer precursor (PLGA_10K_‐PEG_5K_).^69^ Nonetheless, we may conclude that the acoustofluidic device produces much smaller and more uniform NPs than bulk mixing, without significantly changing the ζ‐potential. Although further investigation into the size stability, drug‐loading efficiency as well as ζ‐potential of NPs is required (Note S4 and Figures S7 and S8, Supporting Information), these results have proved that our acoustofluidic synthesis approach can reproducibly synthesize PLGA‐PEG NPs with better physicochemical properties than those prepared by bulk mixing (see Note S5 in the Supporting Information explaining the direct comparison).

**Figure 5:**
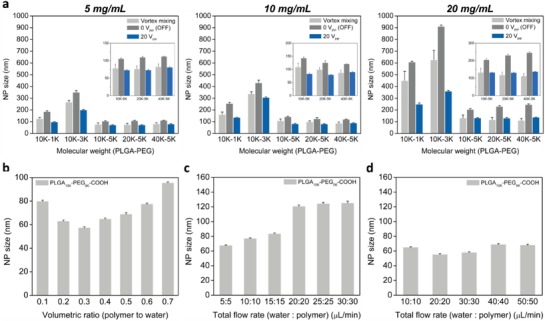
Comparison of average size of PLGA‐PEG NPs synthesized with various parameters. a) Dependence of the average size of synthesized PLGA‐PEG NPs on the precursor concentration and molecular weight, when prepared using different methods: vortex mixing, diffusion‐based mixing (0 V_PP_/OFF), and acoustofluidic device (20 V_PP_/ON). When increasing the precursor concentration, an overall increase in the size of synthesized NPs is observed. Increasing the precursor molecular weight, however, does not guarantee an increase in size in this study. Regardless of the precursor concentration and molecular weight, our acoustofluidic platform consistently yields PLGA‐PEG NPs with the smallest size. Results presented in (a) are obtained under the following working conditions: a driving voltage of 20 V_PP_ and a flow rate of 10 and 1 µL min^−1^ for water and PLGA‐PEG solution, respectively. b) Effect of the volumetric ratio of PLGA‐PEG precursor in bulk solution on the NP size. Overall, increasing the ratio leads to an increase in NP size. c) Effect of the total flow rate on the NP size under the driving voltage of 20 V_PP_. The NP size increases with increasing total flow rate, which can be explained by the compromised mixing performance when increasing the total flow rate. d) Effect of the total flow rate on the NP size under the driving voltage of 50 V_PP_. As a result of using a relatively high voltage, the NP size significantly decreases at all of the total flow rates, compared to those presented in (c). Error bars denote standard deviation from at least three experiments (*n* ≥ 3).

To further highlight the robustness of our synthesis method, we compare the size of NPs produced by acoustofluidic device (20 V_PP_) to those prepared by diffusion‐based mixing (0 V_PP_; OFF) and vortex mixing, using PLGA‐PEG precursors with various molecular weights (MWs) (10K‐1K, 10K‐3K, 10K‐5K, 20K‐5K, and 40K‐5K) at different precursor concentrations (5, 10, and 20 mg mL^−1^). The diffusion‐based mixing is the scenario where the same synthesis device is used but not acoustically activated, removing the acoustic streaming and leaving only diffusion to mix reagents. As the precursor concentration increases, there is an overall increase in size for all the molecular weights tested, irrespective of the synthesis method (**Figure** **5**a). For example, using the 10K‐5K precursor, the acoustofluidic device yields PLGA‐PEG NPs of 73.1 ± 1.58, 83.2 ± 2.43, and 132.6 ± 1.73 nm at the precursor concentrations of 5, 10, and 20 mg mL^−1^, respectively. Similarly, using the 20K‐5K precursor, the acoustofluidic device generates NPs of 73.3 ± 1.66, 79.85 ± 1.33, and 131.6 ± 3.7 nm at the precursor concentrations of 5, 10, and 20 mg mL^−1^, respectively. Similar trends are observed for PLGA‐PEG NPs prepared by the bulk mixing and diffusion‐based mixing. We note, however, that the NP size is not proportional to the molecular weight of the precursor, regardless of the synthesis method. For example, the 10K‐1K and 10K‐3K precursors consistently yield much larger PLGA‐PEG NPs compared to the other precursors, regardless of the synthesis method. This abnormal trend is unexpected and opposite to what has been reported previously.^13^ Typically, when using precursors with large molecular weights for NP synthesis, one would expect to generate NPs with larger size. This phenomenon may be attributed to the source of polymer precursors used in this work: 10K‐1K and 10K‐3K are from one company, while 10K‐5K, 20K‐5K, and 40K‐5K are from the other (Note S3, Supporting Information). Despite this abnormal trend, the acoustofluidic device always produces the smallest NPs with highest reproducibility (i.e., smallest size deviation) and tightest size distribution for all the molecular weights tested, regardless of the precursor concentration (Table S1 and Figure S9, Supporting Information). These results demonstrate that our acoustofluidic device is well suited for polymer‐based spherical NPs synthesis and that it allows for the reproducible synthesis of NPs using various molecular weights of a precursor at varying concentrations.

Adjusting the volumetric ratio of a polymer precursor in a bulk solution could significantly change the size of NPs synthesized. To verify if our platform could alter the size of NPs synthesized by adjusting the volumetric ratio of two solutions, we synthesize NPs by gradually changing the flow rate of the polymer solution (from 1 through 9 µL min^−1^), while keeping the total flow rate in the channel constant (10 µL min^−1^). The ability to completely mix two solutions at varying flow rate ratios is first confirmed by experiments; at the driving voltage of 20 V_PP_, water and FITC can be completely mixed in less than 54 ms at flow rate ratios from 0.1 to 0.9 (Figure S10, Supporting Information). Under the same voltage, our acoustofluidic device can completely mix PLGA‐PEG precursor and water at varying flow rate ratios, thus yielding NPs with average sizes ranging from 57.27 ± 0.98 to 95.37 ± 0.99 nm (Figure 5b). The NP size decreases as the flow rate ratio is decreased from 0.7 through 0.3, and interestingly, increases when the flow rate ratio is further decreased from 0.3 through 0.1. This trend suggests that the flow rate ratio of 0.3 may be the optimal ratio to produce the smallest NPs, but differs from results reported elsewhere,^70^ where the NP size consistently decreased as the volumetric ratio was decreased. The difference in mixing mechanism—active, complete mixing for our synthesis approach versus passive, partial mixing for others—may account for this unexpected trend. Despite this unusual tendency, these results demonstrate that our device can control the NP size by mixing two solutions at different flow rate ratios, and, most importantly, can reveal the independent influence of flow rate ratios on the quality of synthesized NPs without changing mixing time, thus rendering itself capable of identifying an optimal flow rate ratio for a given synthesis condition.

Under a constant driving voltage, the mixing performance of our acoustofluidic device can be degraded because of an increase in the total flow rate, which increasingly suppresses the acoustic streaming.^57^, ^66^ To evaluate the dependence of NP size on the total flow rate, we first synthesize NPs at different total flow rates under a low driving voltage (20 V_PP_), where the flow rate ratio of PLGA‐PEG precursor and water remains at one to simplify the experimental setup. As the total flow rate increases from 10 to 30 µL min^−1^, the NPs grow from 67.3 ± 0.9 to 83.2 ± 1.4 nm and further raising the total flow rate from 30 to 40 µL min^−1^ increases the NP size by nearly 50% to 120.5 ± 2.0 nm; increasing from 40 to 60 µL min^−1^ leads to an increase of less than 5%, to 125.0 ± 2.7 nm (Figure 5c). As the total flow rate increases beyond a certain point (40 µL min^−1^ in this case), the NP size changes insignificantly and becomes independent of the total flow rate; This trend is because, at these higher flow rates, the acoustic streaming induced by the driving voltage of 20 V_PP_ is suppressed significantly and as such, compromises the mixing performance (Figure S11, Supporting Information). Therefore, when synthesizing at relatively high flow rates, we must use higher driving voltages to maintain the mixing performance and therefore, the small NP size. With a driving voltage of 50 V_PP_, for example, the NP sizes are reduced by more than 50% both at the total flow rate of 40 and 60 µL min^−1^; at even higher total flow rates, including 80 and 100 µL min^−1^, the NP size is maintained at 68.8 ± 1.3 and 67.9 ± 1.2 nm, respectively (Figure 5d). It is evident that enhanced acoustic streaming due to the increase in driving voltage retains the mixing performance at high flow rates (Figure S12, Supporting Information), thereby leading to the reduction in the NP size. The ability to synthesize NPs at relatively high flow rates reveals the potential to use our platform for high‐throughput synthesis of NPs/NMs. This result, once again, demonstrates that our platform can tune the properties of synthesized NPs by adjusting the mixing performance, either through adjusting the total flow rate or the power applied to the transducer.

### 2.3 Synthesis of Chitosan NPs

**Figure 6:**
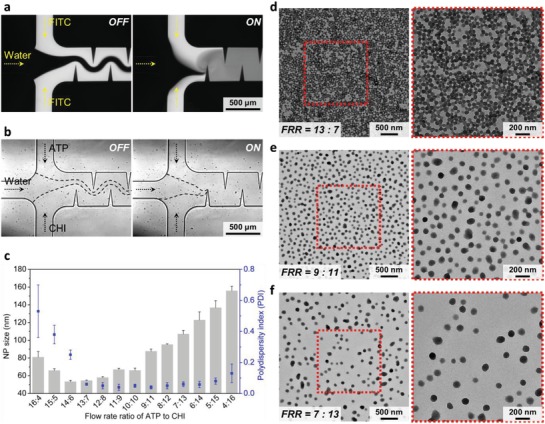
Synthesis of chitosan nanoparticles based on the nanocomplexation (NCP) mechanism using the acoustofluidic device. a) Fluorescent images showing the mixing performance of our acoustofluidic device for mixing three liquids. When the acoustic transducer is off, three solutions establish a laminar flow; once the transducer is activated, three solutions can be rapidly (mixing time ≤54 ms) and completely mixed together after arriving at the second sharp‐edge structure, demonstrating the capability of our micromixer for mixing multiple solutions together at once. b) The acoustofluidic device in operation showing the synthesis of chitosan NPs by mixing together ATP (−), water, and chitosan solutions (+). When the acoustic transducer is off, the three solutions form a laminar flow. Upon activating the transducer (on), the three solutions are completely mixed together, thus achieving the synthesis of chitosan NPs based on the nanocomplexation mechanism. Experiments for (a) and (b) are conducted under the same conditions: a driving voltage of 30 V_PP_ and a flow rate of 10 µL min^−1^ for each solution. c) Average size and polydispersity index of chitosan NPs synthesized at different ATP/chitosan flow rate ratios, where the total flow rate and flow rate of water are set to 30 and 10 µL min^−1^, respectively, and the experiments are conducted under the driving voltage of 30 V_PP_. TEM images of chitosan NPs synthesized at the flow rate ratio of d) 13:7, e) 9:11, and f) 17:3, confirming the size of chitosan NPs synthesized. Error bars denote standard deviation from at least three experiments (*n* ≥ 3).

In NMs/NPs synthesis, rapid yet uniform mixing of multiple liquids may be required. This requirement, however, is quite challenging for many existing microfluidics‐based synthesis methods.^12^, ^13^, ^71^ With an active‐mixing strategy, our platform can easily blend multiple liquids together at different flow rate ratios (Figures S13 and S14, Supporting Information). As an example, our acoustofluidic device can mix three liquids at a flow rate ratio of 1:1:1 (**Figure** **6**a). When the transducer is off, the three liquids form a laminar flow (Figure 6a: Left), whereas when the transducer is on, they are rapidly and completely mixed after passing the first pair of sharp‐edge structures (Figure 6a: Right). Having demonstrated the mixing of three liquids, we then synthesize chitosan NPs by mixing 0.5 mg mL^−1^ chitosan solution, water, and 1 mg mL^−1^ adenosine triphosphate solution (ATP), where the water stream serves to isolate the chitosan solution from the ATP solution to minimize pre‐reaction between them before synthesis. In this demonstration, we fix the total flow rate in the channel and the water's flow rate to 30 and 10 µL min^−1^, respectively, while varying the flow rate ratio of ATP/chitosan solutions from 16:4 through 4:16. When the device is off, chitosan solution, water, and ATP solution form a laminar flow having clear interfaces (Figure 6b: OFF); once activated, our device can completely mix these three solutions together without any fluidic interfaces observed (Figure 6b: ON), thus forming chitosan NPs via ATP‐initiated ionic gelation.^72^

At first glance, each flow rate ratio yields a distinct size distribution, and reducing the flow rate ratio shifts the size distribution toward larger NP sizes (Figure S15a, Supporting Information). By adjusting the flow rate ratio from 16:4 to 4:16, we can produce chitosan NPs with an average size ranging from 53 to 155 nm and a polydispersity index ranging from 0.04 to 0.53 (Figure 6d). When prepared at flow rate ratios from 16:4 to 14:6, the size of synthesized chitosan NPs decreases from 128 to 53 nm as the flow rate of the chitosan solution is raised, along with a wide size distribution (polydispersity index = 0.59 to 0.25). Between the ratios of 14:6 and 4:16, the size increases from 54 to 155 nm as the flow rate of the chitosan solution is increased, but features a narrow size distribution (polydispersity index = 0.06 to 0.16). Chitosan NPs produced at the ratio of 13:7 have a relatively uniform size of 54.6 ± 0.56 nm and a relatively narrow size distribution (polydispersity index = 0.06 ± 0.007), thereby identifying an optimal flow rate ratio to synthesize chitosan NPs using our device. Figure 6d–f shows the TEM images of chitosan NPs prepared at the ratio of 13:7, 9:11, and 7:13, respectively, confirming the size and uniformity of chitosan NPs we prepared. TEM images in the Supporting Information also verify the size and uniformity for chitosan NPs synthesized at other flow rate ratios (Figure S15b–d, Supporting Information). Furthermore, we synthesize chitosan NPs using chitosan/ATP solutions of different concentrations (chitosan = 1.0 mg mL^−1^; ATP = 0.5 mg mL^−1^). After modifying the concentration, we produce the smallest chitosan NPs (49.5 ± 2 nm) at the flow rate ratio of 15:5, suggesting that 15:5 is the optimal flow rate ratio at these given concentrations (Figure S16a, Supporting Information). With this optimal ratio, we then demonstrate that the size of chitosan NPs can be tuned from 42 to 98 nm by adjusting the driving voltage from 0 to 50 V_PP_ (Figure S16b, Supporting Information). These results demonstrate that by using our device, we can identify an optimal flow rate ratio, for given reagent concentrations, to prepare chitosan NPs with desired properties. The results also demonstrate that our platform can synthesize NPs where mixing multiple reagents at different flow rate ratios is required.

### 2.4 Synthesis of Nonspherical NMs and Other Nanocomplexes

**Figure 7:**
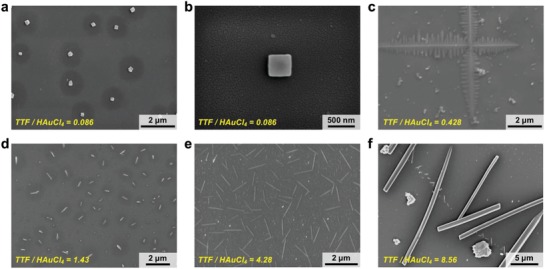
SEM images of nanohybrids synthesized by mixing TTF and HAuCl_4_ solutions at different flow rate ratios. As we gradually increase the flow rate ratio of TTF and HAuCL_4_ solutions from 0.086 through 8.56, the shapes/morphologies of the synthesized TTF–HAuCl_4_ nanohybrids transitioned from a,b) square or rectangular to c) crosses with dendritic nanostructures, to wires of d) several hundred of nanometers in length, e) 0.5–1.5 µm in length, and f) 10–20 µm long. Experiments are conducted under the driving voltage of 30 V_PP_, and the total flow rate is set to 20 µL min^−1^. The detailed flow rates for TTF and HAuCl_4_ solutions are listed in Table S1 in the Supporting Information.

Not only can our acoustofluidic device synthesize polymeric, spherical NPs, but it can also synthesize nonspherical NMs. To demonstrate the capability of synthesizing nonspherical NMs, we synthesize tetrathiafulvalene (TTF)–gold hybrid NMs by mixing TTF and HAuCl_4_ solutions with our device. **Figure** **7** shows the hybrid NMs synthesized with our device at various flow rate ratios of TTF/HAuCl_4_ (see Table S2 in the Supporting Information for detailed flow rates). As the flow rate ratio increases, the morphologies of the hybrid materials synthesized with our device transition from square/rectangular (Figure 7a,b), to 2D dendritic nanostructures (Figure 7c), to nanowires which increase from several hundreds of nanometers in length (Figure 7d), to 0.5–1.5 µm in length (Figure 7e), to microwires of 10–20 µm in length (Figure 7f). When the TTF's volume is much lower than that of HAuCl_4_ in the mixture, square‐like gold crystals are synthesized (Figure 7a,b), because TTF at a relatively low concentration would serve as a capping agent and adsorb on a given plane of gold.^73^, ^74^ Increasing the flow rate ratio by nearly five times can produce 2D dendritic NMs that are entirely different from the gold crystals (Figure 7c); this transformation is due in large part to the interaction of TTF and gold crystallization and the phenomenon of nonequilibrium growth.^75^, ^76^ Additionally, increased TTF's volume may also contribute to the 1D formation of straight “branches” shown in Figure 7c. Further increasing the flow rate ratio to the range where TTF's bulk volume is higher than that of HAuCl_4_ can synthesize wire‐like NMs with size increasing in both the length and diameter as the flow rate ratio increases (Figure 7d–f). In the cases where the volume of HAuCl_4_ is lower than that of TTF, gold crystals could serve as capping agents and then bond to the TTF crystals via Au—S covalent bonds. This phenomenon would allow for the synthesis of TTF‐crystal‐based NMs, and has been found to hinder TTF crystals from growing into multidimensional structures.^77^ Overall, the TTF–Au hybrid NMs synthesized by our device are morphologically similar to those synthesized using passive, diffusion‐limited mixing methods; nevertheless, they are smaller and more uniform in size, thereby demonstrating the capability of synthesizing nonspherical NMs with our acoustofluidic strategy. Even though in this demonstration, we alter the morphology of synthesized NMs only by tuning the flow rate ratio of TTF/HAuCl_4_, we could potentially produce NMs with shapes other than those being presented, by tuning both the mixing time and flow rate ratio. For example, at a given flow rate ratio we may adjust the diameter and length of the TTF/gold hybrid wires synthesized by adjusting the driving voltage (i.e., the mixing time) of the transducer.

**Figure 8:**
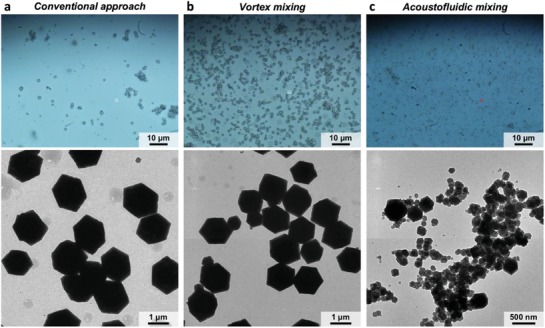
Synthesis of MOF‐based biocomposites (BSA‐@‐ZIF‐8) synthesized by mixing Zn(OAc)_2_⋅2H_2_O, BSA, and HmIM solutions. Optical (top) and TEM (bottom) images of BSA‐@‐ZIF‐8 prepared by a) conventional approach, b) vortex mixing, and c) acoustofluidic device. These results show that our acoustofluidic device can produce BSA‐@‐ZIF‐8 biocomposites that are overall smaller than 500 nm in size, which is smaller than those prepared by either the conventional approach or vortex mixing. The mixing of the Zn(OAc)_2_⋅2H_2_O, water, and HmIM solutions is achieved solely by diffusion‐based and vortex mixing, respectively, for the conventional approach and vortex mixing. The results also demonstrate the capability of our acoustofluidic platform for the synthesis of nonspherical NMs.

As another demonstration of synthesizing nonspherical micro/nanomaterials, we also synthesize metal–organic frameworks with our acoustofluidic device. Recently, MOFs‐based biocomposites have gained considerable attention due to their high surface‐to‐volume ratios and loading capacities, which are beneficial for biosensing/detection applications; however, currently their synthesis still rely on mixing reagents through the free‐diffusion between reagents. In this demonstration, we synthesize BSA‐@‐ZIF‐8, a zeolitic imidazolate framework (ZIF)‐based biocomposite, by completely mixing Zn(OAc)_2_⋅2H_2_O, bovine serum albumin (BSA), and 2‐methylimidazol (HmIM) solutions together through our acoustofluidic strategy. Relative to the conventional approach and bulk mixing, where the Zn(OAc)_2_⋅2H_2_O and HmIM solutions are mixed solely based on diffusion and vortex mixing, respectively, our acoustofluidic device can produce ZIF biocomposites smaller than 500 nm in size, which is at least 50% smaller than those prepared by the conventional approach and bulk mixing (**Figure** **8**). Although further experimentation could reveal more about the ability of our platform to control the size (or shape) of MOFs by adjusting synthesis parameters, such as the HmIM/Zn molar ratio and driving voltage, this result reveals the potential of our acoustofluidic platform to synthesize MOFs with relatively small size. This result also demonstrates, once again, that our platform can synthesize NPs/NMs that require the mixing of multiple reagents.

## 3 Discussion

We present an acoustofluidic platform that can synthesize both spherical and nonspherical NPs/NMs based on an active and complete mixing of reagents enabled by acoustic streaming. Our acoustofluidic platform, by adjusting the strength of the acoustic streaming or the flow rates of reagents, can provide a dynamic switch between three mixing regimes in one single platform: diffusion‐based mixing (when the acoustic field is off); partial/incomplete mixing (when the acoustic streaming is present but not strong enough to achieve complete mixing); and complete and thorough mixing (when the acoustic streaming is relatively strong). These tailored mixing regimes, when working with reagents of different concentrations, molecular weights, or flow rate ratios, offer unprecedented flexibility in controlling the reaction conditions for solution‐based synthesis of NPs/NMs.

In solution‐based synthesis, the mixing time may be the most critical factor governing the formation and resultant physicochemical properties of NPs/NMs. Most existing microfluidics‐based synthesis methods involuntarily dictate their mixing time when adjusting the flow rate ratios of reagents, meaning that both parameters change simultaneously. Our method, by comparison, tunes the mixing time independently by adjusting the strength of acoustic streaming (i.e., the acoustic power). As such, it allows one to independently investigate the influence of the mixing time or the flow rate ratio on the properties of synthesized NPs/NMs. To the best of our knowledge, independently distinguishing these influences has been challenging for existing methods,^31^, ^40^, ^73^ which control the mixing time mainly by altering the flow rate ratio; thus, it remains unclear how a change in the mixing time or flow rate ratio independently affects the properties of synthesized NPs/NMs. We can either keep the flow rate ratio constant and uncover how the mixing time alters the NPs/NMs, or maintain a constant mixing time and reveal how the flow rate ratio affects the synthesized NPs/NMs by altering the flow rate ratio. Such capabilities, in conjunction with the ability to mix multiple liquids at once or in a prescribed order, would enable the synthesis of versatile NPs/NMs with tailored properties. It is notable that our acoustofluidic synthesis approach, at this stage, may still require centrifugation to retrieve the formulated NPs/NMs upon the completion of synthesis; nevertheless, our method does remove the need for phase separation when compared to droplet‐based methods. Additionally, with our device's simple setup and operation, it would be straightforward to integrate our platform with microfluidics‐based nanoparticle separation devices to replace the off‐chip centrifugation.

In this work, we focus primarily on the synthesis of PLGA‐PEG NPs to prove our concept, but we also synthesize other NPs/NMs including chitosan NPs, TTF–Au hybrid NMs, and MOFs‐based biocomposites to demonstrate the wide applicability of our platform. In addition to these NPs/NMs, we also demonstrate the synthesis of lipid/DNA complexes (lipoplexes). Our acoustofluidic platform can reproducibly prepare lipoplexes smaller than 100 nm (Figure S17, Supporting Information), a critical size when determining whether synthesized lipoplexes could be easily internalized by cells.^78^ Further investigation will be needed to fully explore how our platform can precisely tailor the size and morphology of synthesized NPs/NMs and to verify the functionalities of the synthesized NPs/NMs such as enzyme‐ or drug‐encapsulation efficiency.

Our acoustofluidic approach offers an active strategy that can synthesize NPs/NMs in a highly controllable, reproducible manner. It features simplicity in device operation and fabrication, which would allow its integration with existing in‐line NPs/NMs monitoring/characterization systems toward the development of a fully automated synthesis platforms. Our approach enables the fabrication of a wide variety of NPs/NMs with a single device, a feat unobtainable with existing methods. With these above‐mentioned capabilities, our acoustofluidic platform will be invaluable to future NPs/NMs synthesis efforts and explorations.

## 4 Experimental Section

*Materials*: PLGA‐PEG‐COOH with molecular weights of 10, 20, and 40 kDa for PLGA and 5 kDa for PEG were purchased from PolySciTech (Akina Inc., West Lafayette, IN, USA). PLGA‐PEG‐COOH with molecular weights of 10 kDa for PLGA and 1 and 3 kDa for PEG were purchased from Nanosoft Polymers (Winston‐Salem, NC, USA). FITC‐dextran, tetrathiafulvalene, hydrogen tetrachloroaurate (HAuCl_4_), acetonitrile, low‐molecular weight chitosan, adenosine triphosphate, sodium hydroxide (NaOH), glacial acetic acid, purified water (H_2_O), Zn(OAc)_2_ (≥99%), and 2‐methylimidazol (≥99%) were purchased from Sigma‐Aldrich (St. Louis, MO, USA). Lipofectamine 2000 and pcDNA3‐EGFP were purchased from Invitrogen (Carlsbad, CA, USA) and GenScript (Piscataway, NJ, USA), respectively.

*Fabrication of Acoustofluidic Synthesis Device*: The acoustofluidic device, which was composed of a single‐layer PDMS channel, an acoustic transducer, and a glass coverslip, was prepared. The PDMS channel with sharp‐edge structures was fabricated by casting a silicon mold with PDMS. The silicon mold was fabricated by patterning a silicon wafer using standard photolithography and deep ion etching (DRIE), followed by antisticking coating with 1H,1H,2H,2H‐perfluorooctyl‐trichlorosilane (Sigma‐Aldrich, MO, USA). Once the silicon mold was ready, PDMS base agent and curing agent (Sylgard 184, Dow Corning, MI, USA) were mixed at a 10:1 w/w ratio, poured onto the silicon mold, and degassed under vacuum for 30 min. After baking at 65 °C for 60 min, the PDMS cast was completely cured and carefully peeled off from the mold. Two inlets and one outlet were opened on the PDMS channel using a 0.75 mm biopsy punch. The PDMS channel and a 24 × 50 mm^2^ cover glass (Cat. No. 48382‐136, VWR, PA, USA) were treated with O_2_ plasma (BD‐10AS, Electro‐Technic Products, IL, USA) for 10 and 60 s, respectively, followed by bonding the PDMS channel onto the cover glass. After baking the stack of the PDMS channel and cover glass at 65 °C for 24 h, an acoustic transducer (Part no. AB2720B‐LW100‐R, PUI Audio Inc., OH, USA) was bonded next to the PDMS channel on to the cover glass using epoxy (Part no. 84101, Permatex, CT, USA).

*Experimental Setup and Device Operation*: All experiments were carried out on the stage of an inverted microscope (Eclipse Ti‐U, Nikon, Japan) equipped with a fast camera and a charge‐coupled device (CCD) camera. Solutions/reagents were delivered to the device using separate 1 mL syringes (BD, Bioscience, NJ, USA), which were independently controlled by an automated syringe pump (neMESYS, Cetoni, Germany). The acoustic transducer was driven by amplified sinewave signals supplied from a function generator (AFG3011C, Tektronix, USA) and a power amplifier (25A250A, Amplifier Research, USA). An oscilloscope was employed to calibrate the amplified signals prior to each experiment. Each experiment was conducted using at least three separate, independent devices (*n* ≥ 3), in order to prove the reproducibility of the device for NP/NM synthesis.

*NP/NM Characterization*: The size, size distribution, polydispersity index, and surface charge (zeta potential, mV) of synthesized NPs were measured by a dynamic light scattering system (Zetasizer Nano ZSP, Malvern, UK) at room temperature. Viscosity and refraction indices were set equal to those specific of water. The morphologies of synthesized NPs/NMs were examined by transmission electron microscopy and scanning electron microscopy (SEM).

*Synthesis of PLGA‐PEG Nanoparticles*: PLGA‐PEG precursor solutions were prepared by dissolving PLGA‐PEG of different molecular weights in acetonitrile at three concentrations (5, 10, and 20 mg mL^−1^). Unless otherwise indicated, the 10 mg mL^−1^ PLGA_10K_‐PEG_5K_ solution was used in most of the experiments. In the case of acoustofluidic synthesis, the two solutions were injected into the device through two separate inlets at varying flow rates. For results presented in Figure 2, Figure 5a, and Figures S2 and S9 in the Supporting Information, PLGA‐PEG solution and water were injected at the flow rate of 1 and 10 µL min^−1^, respectively, while for results presented in Figure 3, Figure 4, Figures S5 and S6 (acoustofluidic mixing) and Figure S8 in the Supporting Information, PLGA‐PEG solution and water were injected both at the flow rate of 10 µL min^−1^. In the case of bulk synthesis, 50 µL of PLGA‐PEG in acetonitrile was mixed with 50 µL of purified water on a vortex mixer for 1 min. For SEM imaging, specimens were prepared by depositing 20 µL of the sample solution, that is, the synthesized NPs in distilled water, on a tiny slice of silicon wafer; once the synthesized NPs in distilled water were dried out and completely adsorbed on the wafer, a thin layer of gold was sputtered covering the entire surface of the wafer. For TEM imaging, TEM samples were prepared by dispensing a drop of the sample solution on a 300‐mesh carbon‐coated copper grid (Electron Microscopy Science); after drying in the air for ≈30 min, the synthesized NPs were adsorbed on the grid. Then, the NP‐adsorbed grid was negatively stained with sterile‐filtered uranyl‐acetate aqueous solution and incubated for 15 min. The grid was finally washed twice with distilled water and dried at room temperature before imaging.

*Synthesis of Chitosan Nanoparticles*: In this work, chitosan NPs were synthesized by mixing chitosan, purified water, and ATP solutions together at different flow rate ratios. The water phase served to prevent the chitosan solution from reacting with the ATP solution before synthesis. Once activated, the device mixed the three solutions together and thus produced chitosan NPs. To prepare the chitosan solution, first chitosan purification was conducted following the procedures reported elsewhere.^79^ The purified chitosan was dissolved in acetic acid solutions to obtain chitosan solutions of two different concentrations, 1 and 0.5 mg mL^−1^. The ATP solution was prepared by dissolving ATP in ultrapurified water at two different concentrations, 1 and 0.5 mg mL^−1^. It is important to note that for chitosan NP synthesis, pH values for both chitosan and ATP solutions are critical to whether ATP‐initiated ionic gelation can occur and thus produce chitosan NPs. As a result, in this work both of them were carefully adjusted to have a pH value of ≈4, which was reported as a suitable value for the synthesis of chitosan/ATP NPs.^79^ For TEM imaging, TEM samples were prepared by dispensing a drop of the sample solution containing the synthesized chitosan NPs on a 300‐mesh carbon‐coated copper grid for 30 min at room temperature. After the chitosan NPs were adsorbed on the grid, they were negatively stained with sterile‐filtered uranyl‐acetate aqueous solution and incubated for 5 min. The grid was finally washed twice with distilled water and dried at room temperature before imaging.

*Synthesis of TTF–Au Hybrid Materials*: For the synthesis of TTF–Au hybrid NMs, TTF was dissolved in acetonitrile to obtain a 1.1 × 10^−3^ m TTF solution, while HAuCl_4_ was dissolved also in acetonitrile to obtain a 0.27 × 10^−3^ m HAuCl_4_ solution. The two solutions were introduced into the device at varying flow rate ratios, where the total flow rate in the channel remained constant. Detailed flow rate combinations can be found in Table S2 in the Supporting Information. The morphology of the synthesized TTF–Au hybrid materials was examined using SEM immediately after synthesis. SEM specimens were prepared by pipetting a droplet of the sample solution (that is, the synthesized TTF–Au NMs in acetonitrile) on a tiny slice of silicon wafer. After drying out at room temperature for 30 min, the specimens were then imaged by SEM.

*Synthesis of BSA‐@‐ZIF‐8 Biocomposite*: For the synthesis of BSA‐@‐ZIF‐8, the Zn(OAc)_2_ solution was prepared by dissolving Zn(OAc)_2_ in purified water at a concentration of 120 × 10^−3^ m, the HmIM solution was prepared by dissolving HmIM in purified water at a concentration of 1.9 M, and the BSA solution was prepared by dissolving BSA in purified water at a concentration of 1 mg mL^−1^. In the case of acoustofluidic synthesis, the three solutions were injected into the device through three separate inlets each at a flow rate of 15 µL min^−1^ and the mixture was collected over a given time interval (13–15 min) to reach a sample volume of ≈600 µL. The device was activated at the driving frequency of 4.0 kHz and driving voltage of 30 V_PP_. In the case of standard synthesis, 200 µL BSA solution was first mixed with 200 µL HmIm solution in a 1.5 µL microcentrifuge tube via brief mixing (i.e., manually but gently shaking the tube) for 10 s. The resultant mixture was then mixed with 200 µL Zn(OAc)_2_ solution via brief mixing for 10 s. In the case of bulk synthesis, 200 µL BSA solution and 200 µL HmIm solution were first added in a 1.5 µL microcentrifuge and mixed together via vortex mixing for 10 s. Then, 200 µL Zn(OAc)_2_ solution was added into the same tube and mixed with the previous mixture via vortex mixing for 10 s. The samples from the three cases were all followed by a 24 h incubation at room temperature. After incubation, they were purified first by centrifugation at 600 rpm for 5 min and rinsed with purified water for three times. Following purification, the purified product was resuspended in 1 mL purified water for TEM characterization. TEM specimens were prepared by immersing the copper grid in the purified sample solution (that is, purified BSA‐@‐ZIF‐8 in distilled water) for 10 s, followed by evaporation at room temperature for 30 min.

*Synthesis of Lipid/DNA Complexes*: For the synthesis of lipid/DNA complexes, lipid solution was prepared by dissolving lipofetin in purified water at a concentration of 20 µg mL^−1^, and DNA solution was prepared by dissolving pcDNA‐EGFP in water at a concentration of 10 µg mL^−1^. In the case of acoustofluidic synthesis, the two solutions were injected into the device through two separate inlets both at a flow rate of 10 µL min^−1^. To perform the synthesis, the device was driven at the driving frequency of 4.0 kHz and driving voltage of 30 V_PP_. In the case of bulk synthesis, 50 µL of lipid solution was mixed with 50 µL DNA solutions on a vortex mixer for 1 min.

## Conflict of Interest

The authors declare no conflict of interest.

## Supporting information

Supplementary
