# Supplementary material for: Acoustofluidic Synthesis of Particulate Nanomaterials
Source: Adv Sci (Weinh). 2019 Aug 27;6(19):1900913. doi: 10.1002/advs.201900913 (PMC6774021; doi:10.1002/advs.201900913)
Supplement: Supplementary file 1 — Supplementary [file ADVS-6-1900913-s001.pdf]

Copyright WILEY-VCH Verlag GmbH & Co. KGaA, 69469 Weinheim, Germany, 2019.

ADVANCED SCIENCE
Open Access

Supporting Information

for *Adv. Sci.,* DOI: 10.1002/advs.201900913

Acoustofluidic Synthesis of Particulate Nanomaterials

*Po-Hsun Huang, Shuaiguo Zhao, Hunter Bachman, Nitesh Nama, Zhishang Li, Chuyi Chen, Shujie Yang, Mengxi Wu, Steven Peiran Zhang, and Tony Jun Huang**

Copyright WILEY-VCH Verlag GmbH & Co. KGaA, 69469 Weinheim, Germany, 2019.

Supporting Information

Acoustofluidic synthesis of particulate nanomaterials

*Po-Hsun Huang,[a‡] Shuaiguo Zhao,[a‡] Hunter Bachman,[a] Nitesh Nama,[b] Zhishang Li,[a] Chuyi Chen,[a] Shujie Yang,[a] Mengxi Wu,[b] Steven Peiran Zhang,[a] and Tony Jun Huang[*a]*

‡These authors contributed equally.

*Corresponding author

Supplementary Note 1: Estimation of the mixing time and mixing index

The mixing time of our acoustofluidic device is estimated by the following equation,

$$\tau_{mix} = \frac{L_{mix}}{V_{mean}}$$

where τ_{mix}, L_{mix}, and V_{mean} are the mixing time, mixing distance (that is, the distance from unmixed to completely mixed regions), and mean fluid velocity, respectively. The mixing distance is

experimentally determined, while the mean fluid velocity is calculated by dividing the total flow rate in the channel by the cross-sectional area of the channel part where the sharp-edge structures are constructed (300 μm × 100 μm). Because the spacing between each sharp-edge structure remains constant in our acoustofluidic device, as shown in **Figure 1**b, the mixing distance is determined using the spacing as a basic distance. Taking **Figure S1**c as an example, we observe that the two fluids, which are injected both at 10 μL/min, are completely mixed after passing the third sharp-edge structure, that is, before traveling to the fourth sharp-edge structure. In this case, the mixing distance is estimated to be less than or equal to 900 μm, and the corresponding mixing is thus calculated to be less than or equal to 80 ms.

The mixing index (*M*) is calculated by the following equation:

$$M = \sqrt{\frac{1}{n}\sum_{i=1}^{n}\left(\frac{I_i - I_m}{I_m}\right)^2}$$

where n, I_i, and I_m are the total number of points sampled along a line or within a region, gray scale value of a given point from that line or region, and average gray scale value of that line or region. From this equation, we know that the mixing index is the standard deviation of gray scale values for that line of region. A mixing index of 0.5 represents completely unmixed fluids, while a mixing index of 0.0 represents competently mixed fluids. Typically, a mixing index of 0.1 is specified as the lower bound for acceptable complete mixing; in other words, mixing indices falling into the range between 0.0 and 0.1 can be regarded as achieving complete mixing of fluids. To be stricter on the mixing index to ensure the maximized mixing performance for nanoparticle synthesis, work we choose a mixing index of 0.05 as the lower bound for acceptable complete mixing of fluids. Taking Figure S1a and S1c as examples, we characterize the mixing index along the dashed lines positioned after the third sharp-edge structures; the mixing index is calculated to be 0.012 and 0.45 for the mixing performance obtained at 3.0 and 4.0 kHz, respectively. Please see Supplementary Note 2 for the

detailed procedures to determine the resonant frequency and optimal length of the sharp-edge structure. Briefly, we determine the resonant frequency and optimal length by evaluating the strength of acoustic streaming and the mixing performance.

Supplementary Note 2: Design optimization of the acoustofluidic synthesis platform

Since PLGA-PEG NPs are synthesized by actively and completely mixing water and polymer solution together, as opposed to passively and partially mixing reagents, the mixing performance of our acoustofluidic device predominately affect the size and uniformity of synthesized NPs. To optimize the mixing performance of our acoustofluidic device, we then investigate the size dependence of PLGA-PEG NPs on the design parameters of our device including the driving frequency of the transducer, and the length and number of the sharp-edge structures (Figure 1b). During this optimization process, the PLGA-PEG solution and water were infused into the channel, respectively, at the flow rates of 1 and 10 μL/min, unless otherwise specified.

Among the device parameters to be tested, a driving frequency that induced the strongest acoustic streaming was the first to identify. To do so, we first employed an acoustofluidic device with only four pairs of sharp-edge structures of 300 μm long, and examined its mixing performance at various driving frequencies by sweeping the frequency with a 0.1 kHz increment from 1 kHz to 50 kHz. Using the mixing of FITC and water as an indicator, we observed that when the device was activated at the frequency of 4.0 kHz, a rapid, complete, and homogeneous mixing was achieved (**Figure S2**), suggesting that 4.0 kHz may be the optimal working frequency for rapid, complete mixing. To further verify if 4.0 kHz is the optimal frequency for synthesis experiments, we then used this frequency, along with other frequencies, to synthesize PLGA-PEG NPs. Dynamic light scattering analysis shows that when our device worked at 4.0 kHz, PLGA-PEG NPs could be synthesized with the narrowest size distribution, the lowest average polydispersity index of 0.13 ± 0.01 and the smallest Z-average diameter of ~ 88.3 ± 0.6 nm (**Figure S3**a and S3b). This result confirms that 4.0 kHz is the

optimal frequency to be adopted in our devices for following experiments. More importantly, the small standard deviations in polydispersity index (±0.01) and NPs size (±0.06 nm) across different experiments (*n* ≥ 3) has demonstrated the reproducibility and robustness of our acoustofluidic synthesis platform.

After finalizing the working frequency, we then investigate how the length (L) of sharp-edge structure can affect the mixing performance and therefore, the NPs size. As a comparison, we also flow the water and PLGA-PEG solution through an empty channel (*i.e.*, without any sharp-edge structures; *L* = 0 µm), verifying if our acoustofluidic device does achieve rapid mixing and thereby, decreases the NPs size. Figure S3c and S3d show, respectively, the size distribution and average size of PLGA-PEG NPs synthesized using acoustofluidic devices with sharp-edge structures of various lengths. When an empty channel is used, no acoustic streaming could be induced upon activating the device, and the synthesis of PLGA-PEG NPs occurs based entirely on slow, diffusion-limited mixing; hence, PLGA-PEG NPs with a wide size distribution and a relatively large average size are produced. As we increase the length from 0 to 300 µm, the size distribution becomes narrower and the average NPs size is reduced from 156.5 ± 2.2 nm to 95.3 ± 1.4 nm. The results suggest that increasing the length of sharp-edge structure substantially enhances the acoustic streaming effect (**Figure S4**).[58] This enhanced acoustic streaming can significantly improve the mixing performance, thereby leading to the decrease in NPs size. Based on this result, the acoustofluidic device with sharp-edge structures of 300 µm long is employed for following experiments.

Supplementary Note 3: Synthesis reproducibility of polymeric NPs

In an effort to leverage our technology and explore how individual synthetic parameters affect the NPs/NMs properties, one would seek to have the most consistent results within individual experiments. Using the same batch of precursor, our acoustofluidic method can reproducibly yield PLGA-PEG NPs with a size variation of ± 1 nm and a variation of ± 0.02 in polydispersity index among

independent experiments (Figure 3d). This degree of reproducibility can also be achieved for samples produced using another batch of precursor (**Figure S5**), thus demonstrating the reliability and robustness of the acoustofluidic synthesis and agreeing with what was concluded elsewhere.[29] The second batch of precursor, however, produced NPs that are overall smaller than those yielded using the first batch. This result implies that using different batches of precursor, even when purchased from the same company, may not guarantee reproducibility among experiments, even with the same synthesis conditions. We found a similar phenomenon in the synthesis of PLGA-PEG NPs using precursors of different molecular weights sourced from two different companies (**Figure 5**a). Theoretically, the size of synthesized NPs should increase as the molecular weight of precursor is increased; however, this trend was not observed in our experiments, and the change in the NPs size was insignificant as we increased the molecular weight. Because of this phenomenon, we believe that the source of chemical reagents used to synthesize NPs/NMs strongly influences the final qualities of NPs/NMs. Alternatively, we may attribute the lack of an increase in the NPs size with increased molecular weights to our mixing mechanism. It is possible that our acoustofluidic device achieves complete solvent exchange before additional polymers adsorb on NPs that have already formed, or that our acoustofluidic device circulates the mixture so rapidly that additional polymers have insufficient time to aggregate on the surface of existing NPs. To test this hypothesis, we could explore polymers from additional companies and compare the size and uniformity of synthesized NPs. Nevertheless, our acoustofluidic mixing consistently yields smaller and more uniform NPs than vortex mixing, regardless of polymer batch, molecular weight or molecular concentration.

Supplementary Note 4: Size stability and drug loading of PLGA-PEG NPs synthesized

To further evaluate the quality of the PLGA-PEG NPs synthesized by our platform, we examine their size stability upon storage. For size stability, we first confirm if the NP size changes upon adding 50 μL water into the vial containing 50 μL resulting mixture immediately after the synthesis, under

different synthesis conditions. In other words, we examine the size stability of the synthesize NP in water. No difference in size is observed between before and after adding water for all the conditions tested, except for one condition where the device is switched off (0 V_{PP}) and the water and polymer solution are injected at the flow rate of 10 μL/min for both. This specific condition is the same as that presented in Figure 3d (0 V_{PP}), and under this condition, slow mixing takes place solely based on diffusion and yields larger NPs; upon adding more water, NP assembly continues in a slow time scale, which allows for the aggregation of the polymers onto the NPs already synthesized and therefore, increases the NP size after adding water (**Figure S7**a). Knowing the size change upon adding water for different conditions, we then characterize the size stability for the same samples over a 96-hour period. Likewise, the change in NP size is negligible after 48 and 96 hours for all the conditions, except for that specific condition mentioned above (Figure S7b). For instance, the size is reduced from 71.3 ± 0.9 nm to 68.2 ± 1.0 nm and then to 66.8 ± 1.5 nm, respectively, after 48 hours and 96 hours, for the NPs prepared by acoustofluidic mixing at the flow rate ratio of 10:10. These results suggest that the NPs synthesized by our acoustofluidic device are stable in size when suspended in a solution containing a large fraction of water, and proves that the acoustofluidic device (*e.g.*, under 30 V_{PP}) yields NPs that are much smaller than those prepared based solely on diffusion-based mixing (0 V_{PP}; acoustics OFF).

To verify if the NPs size increased after drug loading, we use docetaxel (Dtxl) as a model drug for encapsulation and premix it with the PLGA-PEG solution. Upon loading docetaxel, the NPs size changes within only ± 2 nm, irrespective of the driving voltage (**Figure S8**); the change is less significant and can be neglected, which disagrees with those reported in literatures. This insignificant change in size, on one hand, may suggest that we can yield drug-encapsulated NPs without dramatically changing their sizes. The acoustofluidic device vigorously circulates the mixture *via* the acoustic streaming, thereby facilitating the uniform bonding of hydrophobic docetaxel to hydrophobic PLGA cores and potentially, yielding smaller NPs encapsulated with docetaxel. On the

other hand, the negligible size change can also suggest that no docetaxel is loaded into the NPs, given intrinsically low zeta-potential of this $PLGA_{10K}$-PEG_{5K} precursor, as presented in **Figure S6**. Since NPs with low zeta potentials are highly difficult to load with drug, their sizes will not grow significantly upon loading drug. Nonetheless, more characterizations such as drug encapsulation efficiency should be carried out to confirm the applicability of the NPs we synthesized.

Supplementary Note 5: Comparison on the time allowed for mixing in two differnt cases

In the acoustofluidic experiments, the total flow rate in the channel is 20 µL min^{-1} (unless otherwise indicated), which corresponds to a fluid velocity of ~11.1 mm sec^{-1}. Given that the channel length (measured from the first sharp-edge structure to the last one for the device with 13 pairs of sharp-edge structures) is 7.5 mm, the time allowed for mixing liquids in the channel is thus about ~ 675 ms for the acoustofluidic experiments, which is much shorter than that (1 minute) in the vortex experiments. In other words, we will need to fabricate an acoustofluidic device with a channel length of ~ 666 mm to directly compare the mixing performance with the vortex setup, which can be very impractical. Even though 1 minute is allowed for mixing reagents in the vortex experiments, the reproducibility and the physicochemical property (*e.g.*, the size and uniformity) of the generated NPs/NMs are incomparable to those generated by the acoustofluidic experiemtns. This observation suggests that the acoustofluidic setup is capable of rapidly yet homogeneously mixing reagents, therefore enabling the rapid exchange of solvents and reproducibly yielding smaller NPs (referred to Figure S4).

Other than the time allowed for mixing, the strength of the vortexing (from the vortex mixer) and the acoustic streaming need to be taken into account for a direct comparision on the synthesis performance between the two group. Quantitatively matching the strength of the aocustic streaming with the power of the vortex mixer, however, is practically and technically challenging. We would like to mention that in the vortex experiments, the power (*i.e.*, the speed) of the vortex mixer is always

set to maximum so that we can guarantee the mixing of reagents in the centrifugre tube is performed under the strongest vortexing. The result shows that even with the vortex power set to maximum for 1 minute, the vortex mixer still produces NPs that are larger and less uniform than those produced by the acoustofluidic device, in an unreproducible manner. Although we are unable to match the strength for a direct comparison, we are confident that the acoustofluidic device does outperform the vortex mixer in generating small-size, uniform NPs.

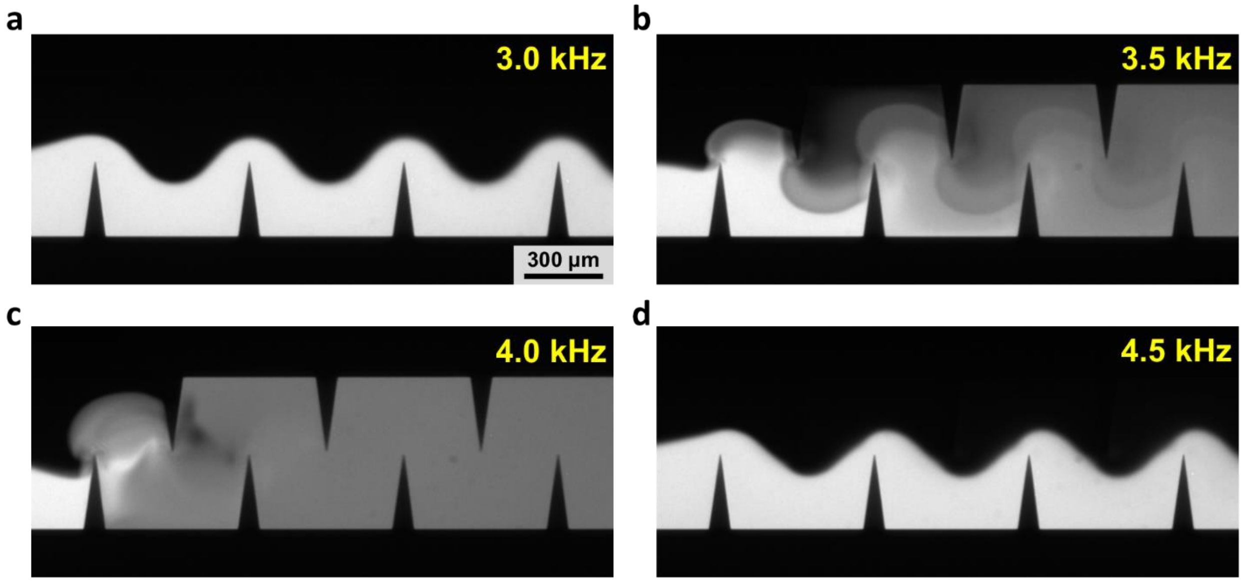

Figure S1. Fluorescent images showing the mixing performance of the acoustofluidic device driven at four different frequencies (**a**) 3.0 kHz, (**b**) 3.5 kHz, (**c**) 4.0 kHz, and (**d**) 4.5 kHz. These results reveal that when activated at the frequency of 4.0 kHz, our acoustofluidic device can rapidly and completely mix water and fluorescent dye together with a mixing index (M) of 0.012, before the two solutions reach the fourth sharp-edge structure, which corresponds to a mixing time of ~80 ms. Based on these results, an optimal driving frequency of 4.0 kHz is identified for our device. The two solutions are injected both at a flow rate of 10 μL/min and the driving voltage applied is 30 V_{PP}.

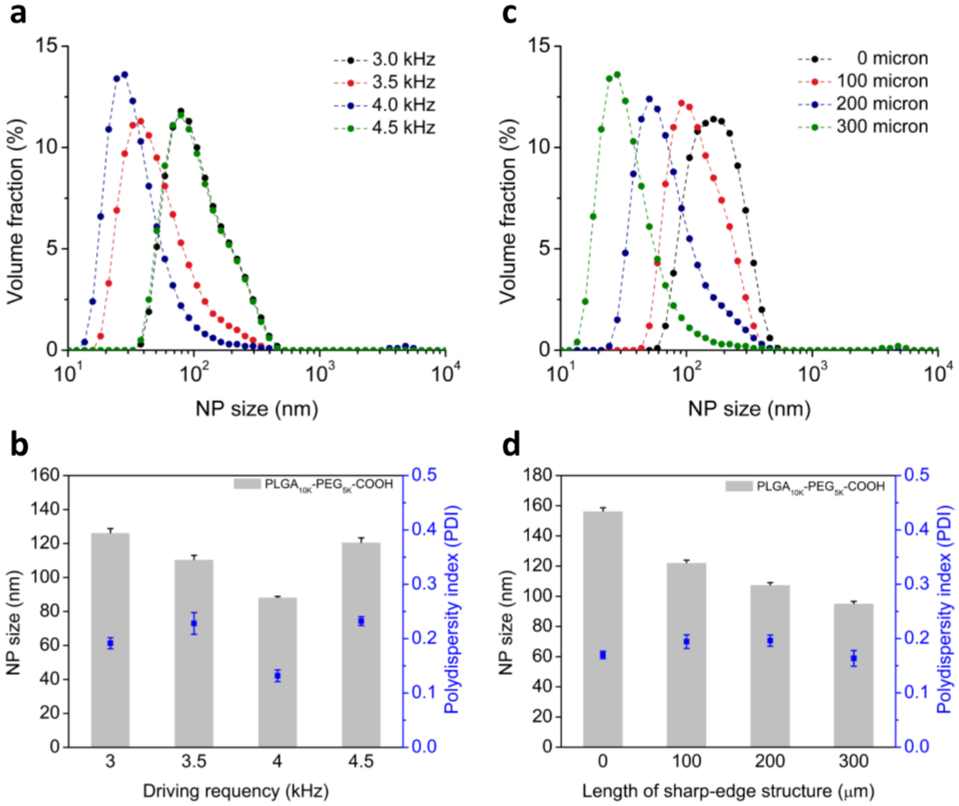

Figure S2. Determination of an optimal driving frequency and an optimal number of sharp-edge structures for our acoustofluidic device. (**a**) Size distribution and (**b**) average size for PLGA-PEG NPs produced with the acoustofluidic device activated at varying frequencies. When the device is driven at a driving frequency of 4.0 kHz, PLGA-PEG NPs could be synthesized with the tightest size distribution (polydispersity index = 0.13 ± 0.01) and the smallest average diameter of ~ 88.3 ± 0.6 nm. (**c**) Size distribution and (**d**) average size for PLGA-PEG NPs synthesized using acoustofluidic devices

with sharp-edge structures of varying lengths. As the length of sharp-edge structures is increased from 0 to 300 µm, the size distribution becomes narrower and the average NPs size is reduced from 156.5 ± 2.2 nm to 95.3 ± 1.4 nm. The results suggest that increasing the length of sharp-edge structures substantially enhances the acoustic streaming effect, which, in turn, improves the mixing performance and results in a reduction in the NPs size. Experiments for these results are carried out using acoustofluidic devices with eight sharp-edge structures, under the following conditions: a driving voltage of 20 V_{PP} and a flow rate of 1 and 10 µL/min, respectively, for the water and polymer solution. Error bars denote standard deviation from at least three experiments (n ≥ 3).

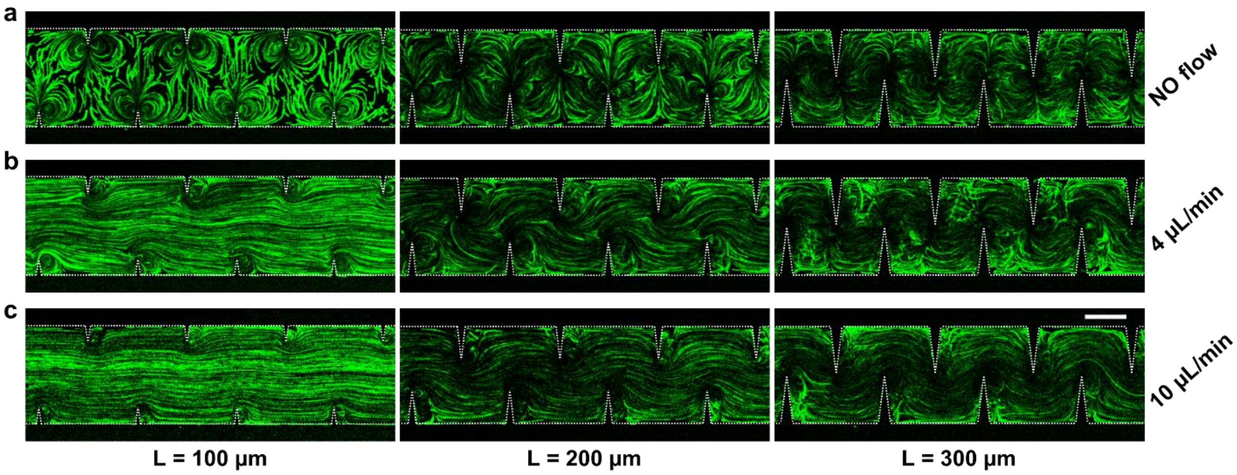

Figure S3. Fluorescent images showing the acoustic streaming induced by sharp-edge structures with varying lengths. (**a**) In the absence of a background flow (No flow), sharp-edge structures with varying lengths can all induce acoustic streaming, when acoustically activated. (**b**) In the presence of a background flow (4 µL/min), sharp-edge structures with varying lengths can still induce acoustic streaming; however, the acoustic streaming generated is suppressed by the background flow. (**c**) Once the background flow further increases to 10 µL/min, the acoustic streaming is significantly suppressed, especially for the device with sharp-edge structures of 100 µm long. The acoustic streaming generated by longer sharp-edge structures is less suppressed. Increasing the length of sharp-edge structures can significantly enhance the acoustic streaming. Experiments for these results are conducted using acoustofludic devices with eight sharp-edge structures, under the following conditions: a driving voltage of 25 V_{PP} and a flow rate ratio of water/FITC of 1. Scale bar: 300 µm.

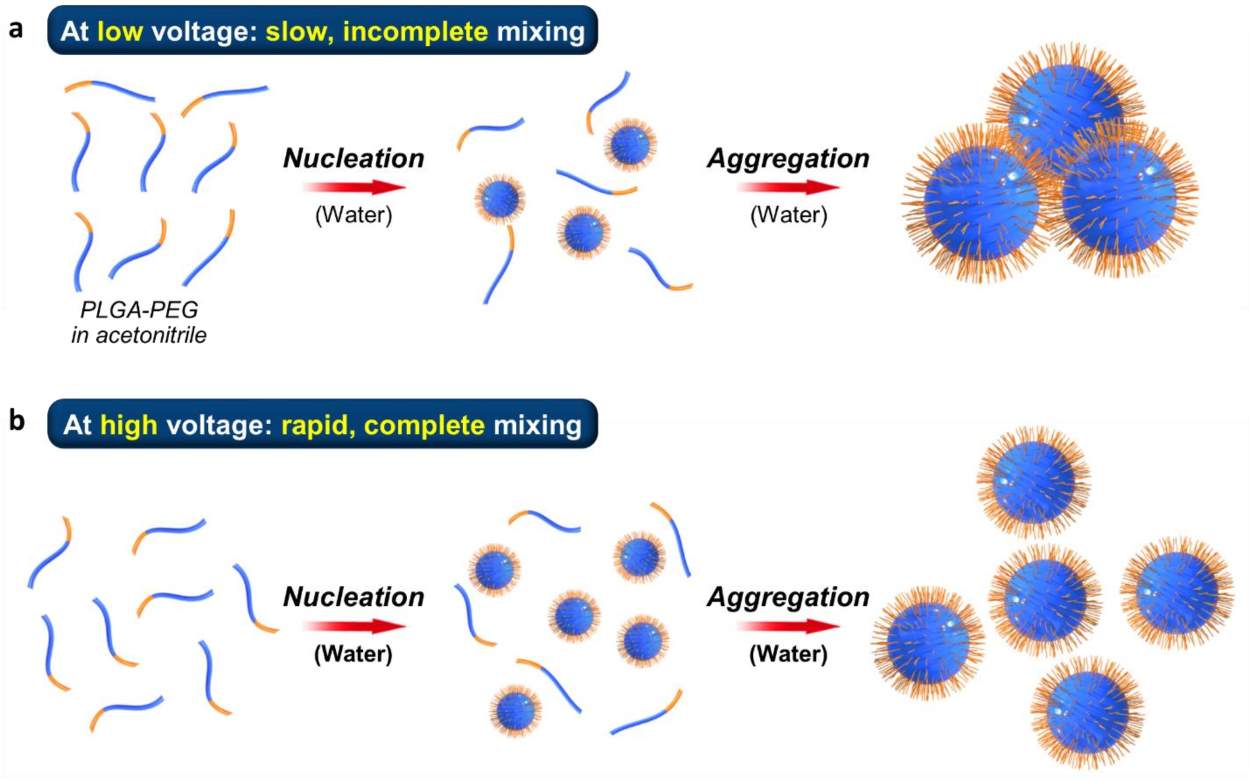

Figure S4. Mechanism of self-assembly of PLGA-PEG nanoparticles based on acoustofluidic mixing. (**a**) At a lower driving voltage, the mixing of water and PLGA-PEG precursor is slow and incomplete, and so is the solvent exchange, where the time scale to achieve complete solvent exchange (τ_{mix}) is longer than the time scale for polymers to start aggregating into nanoparticles (τ_{agg}). Under this condition, polymers nucleate fewer nanoparticles (seeds) and tend to adsorb on these seeds, thus producing

larger nanoparticles. (**b**) At a higher driving voltage, the solvent exchange is rapid and is completed even quicker than τ_{agg}. Under this condition, polymers form more seeds and cannot easily insert or aggregate onto existing seeds, thus producing smaller nanoparticles.

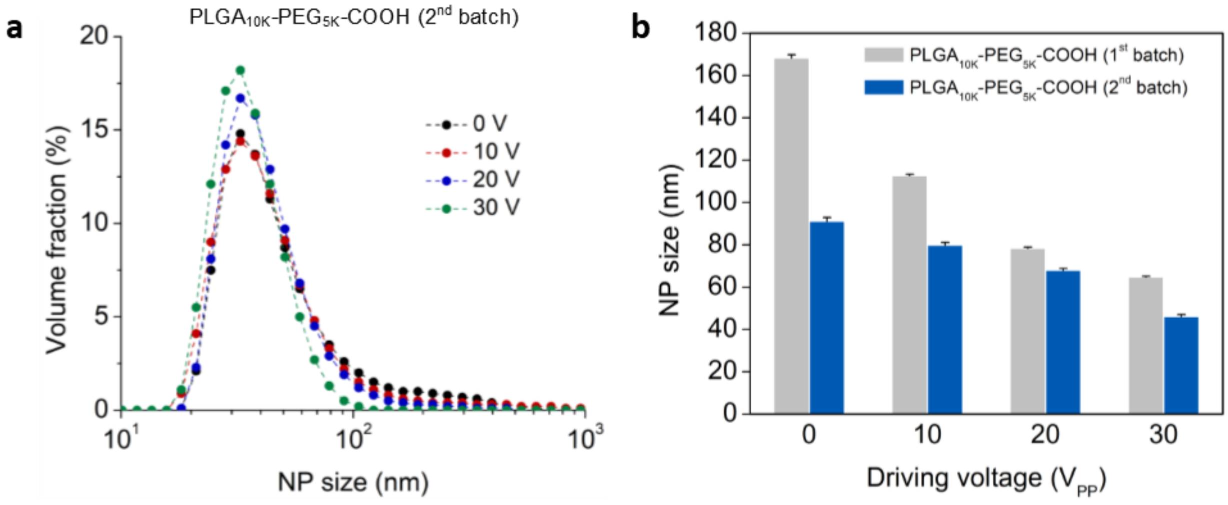

Figure S5. Size distribution and average size of NPs synthesized using another batch of $PLGA_{10K}$-PEG_{5K} precursor. (**a**) Similar to the result represented in Fig. 3c, the volume fraction is raised as we increase the driving voltage, and the size distribution moves towards smaller sizes (though slightly). (**b**) Using this new batch, our platform produces NPs with sizes ranging from 79.9 ± 1.22 to 46.1 ± 0.96 nm and polydispersity indices ranging from 0.26 ± 0.019 to 0.11 ± 0.005, as the driving voltage is increased from 10 to 30 V_{PP}. These results demonstrate that our platform can reproducibly synthesize NPs with a size deviation of ± 1.5 nm and a deviation of ± 0.02 in polydispersity index. The second batch of PLGA-PEG precursor, however, yields NPs that are overall smaller than those produced using the first batch. Error bars denote standard deviation from three experiments (n = 3).

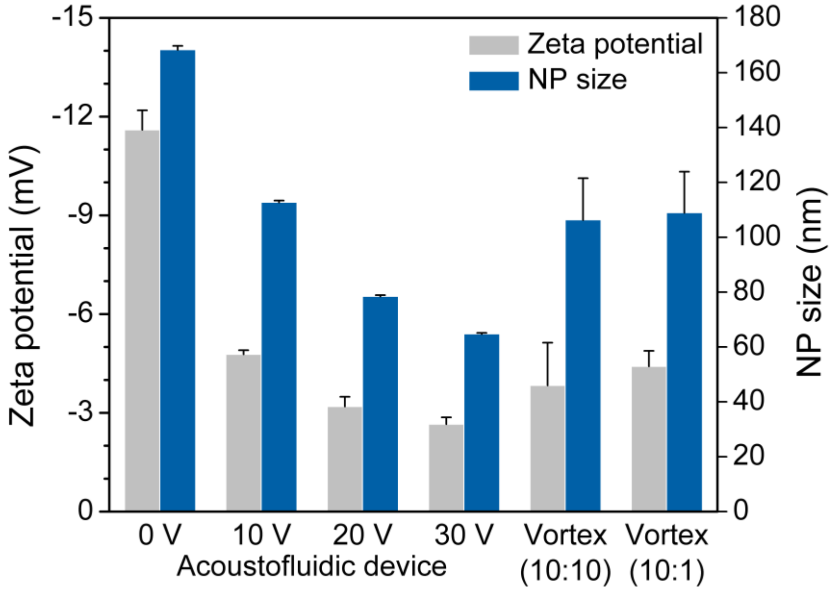

Figure S6. Zeta potential (ζ-potential) for $PLGA_{10K}$-PEG_{5K} NPs prepared by acoustofluidic device and vortex mixing (bulk). When the driving voltage is increased, the NPs size is decreased, along with a decrease in zeta potential. Compared with bulk mixing at the flow rate ratios of 10:10 and 10:1, overall the acoustofluidic device yields smaller nanoparticles without significantly changing the surface charge of synthesized nanoparticles. Error bars denote standard deviation from three experiments (n = 3).

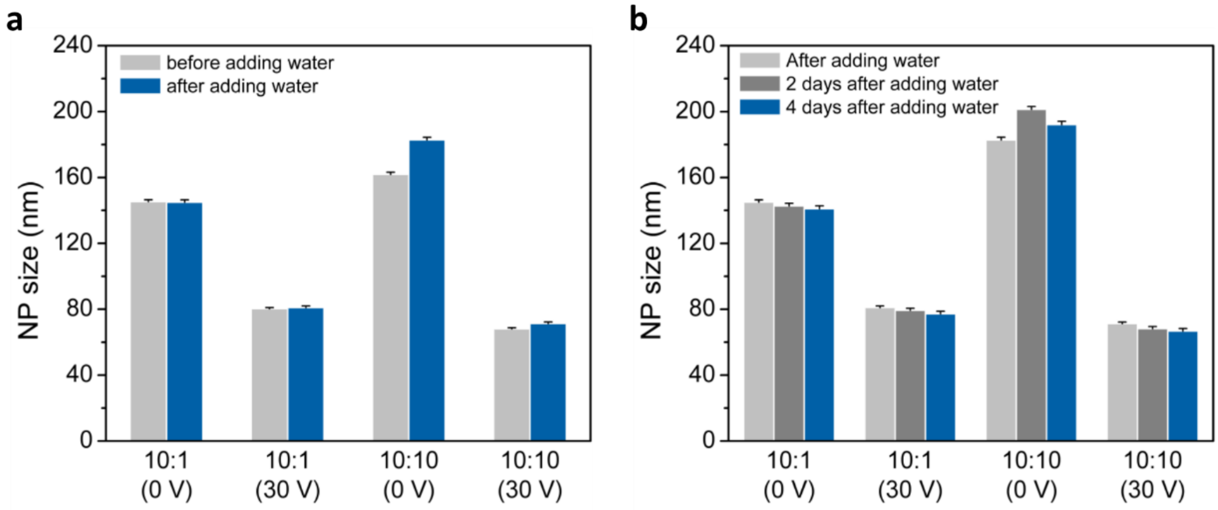

Figure S7. Size stability for the PLGA-PEG NPs synthesized by the acoustofluidic device. The size stability is evaluated upon adding 50 μL water into a vial containing 50 μL resultant mixture immediately after the synthesis, under different synthesis conditions. (**a**) No difference in size is observed between before and after adding water for all the conditions tested, except for one condition where the device is switched off (0 V_{PP}) and the water and polymer solution are injected at an equal flow rate (10 μL/min). These results suggest that with its active, complete mixing, our acoustofluidic platform may minimize the amount of unreacted reagents. (**b**) The change in NP size is negligible after 48 and 96 hours for all the conditions, except for that specific condition mentioned in (**a**). These results indicate that the NPs synthesized by our acoustofluidic device are stable in size, when suspended in a solution containing a large fraction of water, and proves, once again, that our acoustofluidic device (*e.g.*, under 30 V_{PP}) yields NPs that are much smaller than those prepared solely

by diffusion-based mixing (0 V_{PP}). Error bars denote standard deviation from three experiments (n = 3). See **Supplementary Note 4** for details.

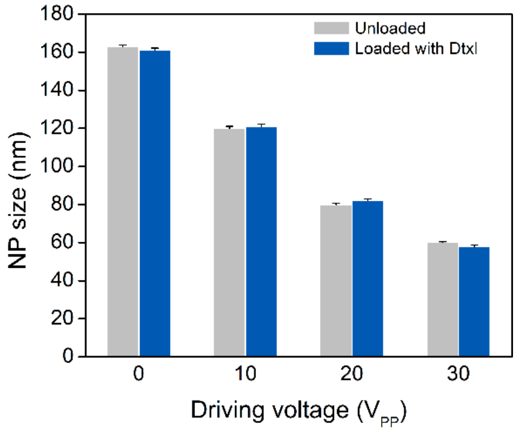

Figure S8. Size change for PLGA-PEG NPs loaded with docetaxel (Dtxl). Upon loading docetaxel, the NPs size changes insignificantly (± 2 nm), irrespective of the driving voltage. This insignificant change in size, on one hand, may suggest that we can yield drug-encapsulated NPs without dramatically changing their sizes. The acoustofluidic device vigorously circulates the mixture *via* the acoustic streaming, thereby facilitating the uniform bonding of hydrophobic docetaxel to hydrophobic PLGA cores and potentially, yielding smaller NPs encapsulated with docetaxel. On the other hand, the negligible size change can also suggest that no docetaxel is loaded into the NPs, given intrinsically low zeta-potential of this $PLGA_{10K}$-PEG_{5K} precursor, as presented in **Figure S6**. See **Supplementary Note 4** for details.

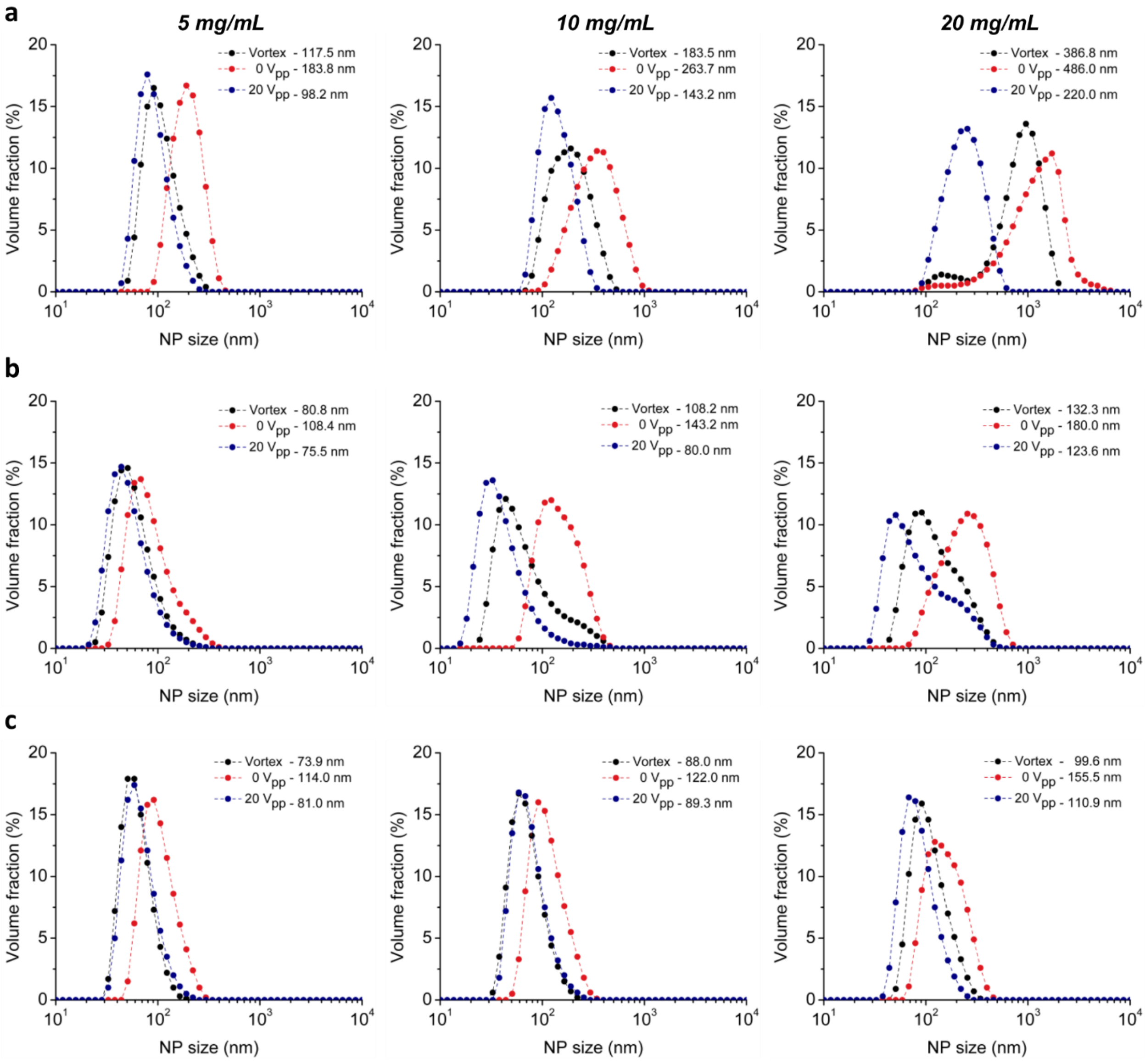

Figure S9. Size distributions of the NPs synthesized using PLGA-PEG precursors of different molecular weights at precursor concentrations of 5 mg/mL, 10 mg/mL, and 20 mg/mL by acoustofluidic device, diffusion-based mixing, and bulk mixing. As a brief comparison, only size distributions yielded from using (**a**) $PLGA_{10K}$-PEG_{1K}, (**b**) $PLGA_{10K}$-PEG_{5K}, and (**c**) $PLGA_{40K}$-PEG5K are presented here. Compared to diffusion-based mixing and bulk mixing, the acoustofluidic device consistently yields smaller PLGA-PEG NPs with narrower size distributions and higher volume fraction for all the molecular weights tested, regardless of precursor concentration.

a

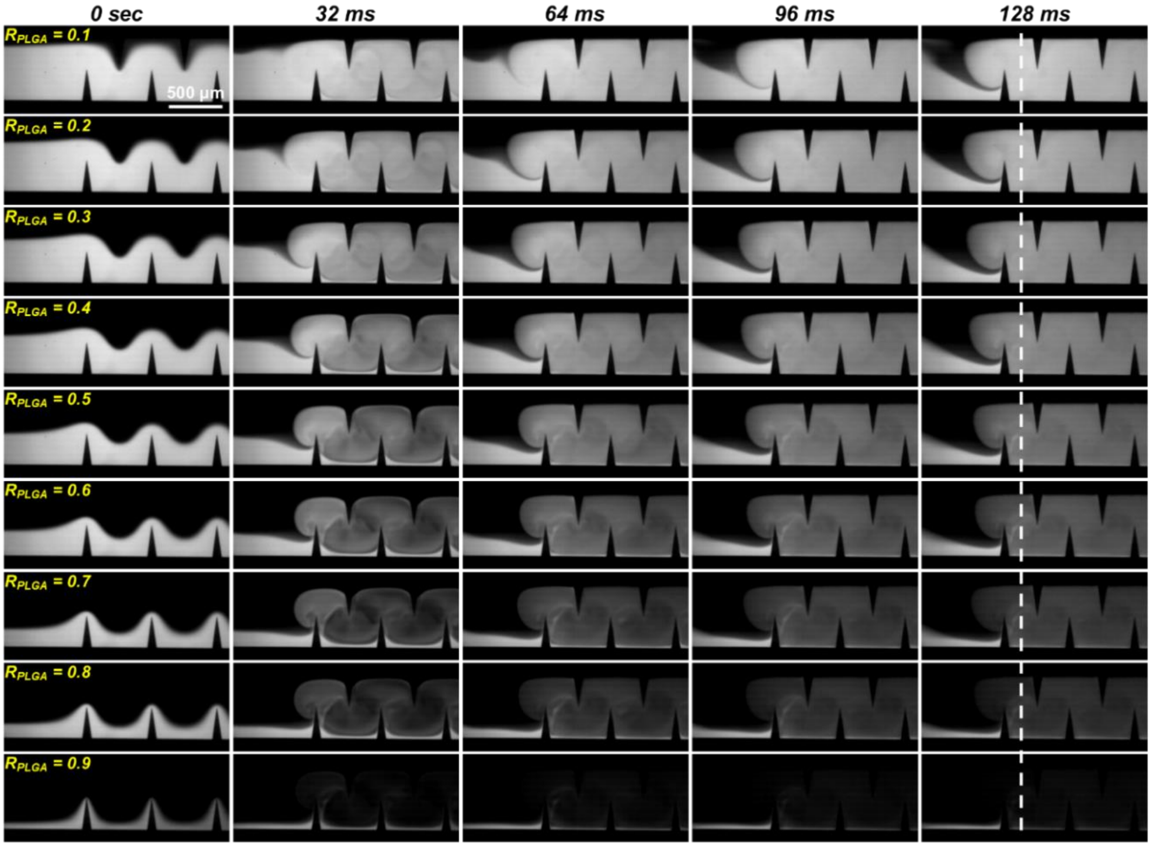

b

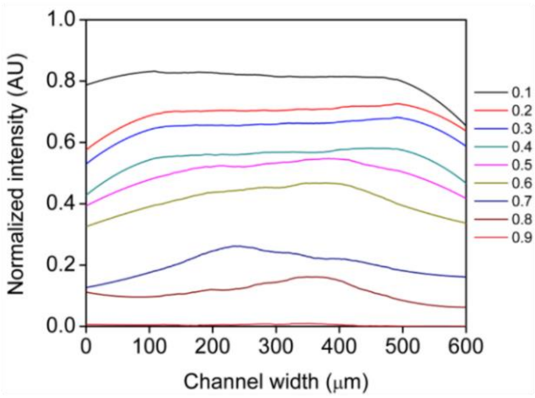

c

R_{PLGA}	Mixing index (at dash line)	Mixing distance (µm)	Mixing time (ms)
0.1	0.040	< 300	< 54
0.2	0.036	< 300	< 54
0.3	0.035	< 300	< 54
0.4	0.038	< 300	< 54
0.5	0.045	< 300	< 54
0.6	0.047	< 300	< 54
0.7	0.050	< 300	< 54
0.8	0.034	< 300	< 54
0.9	0.013	< 300	< 54

Figure S10. Mixing performance presented by mixing water and FITC at different volumetric ratios (water to FITC) from 0.1 to 0.9 under 20 V_{PP}. (**a**) Fluorescent images showing the mixing behavior at different volumetric ratios. (**b**) Corresponding concentration profiles at different volumetric ratios along the white dash line in (**a**). (**c**) Calculation of mixing time at different volumetric ratios. These results demonstrate that our acoustofluidic device can rapidly and completely mix two solutions at different volumetric ratios with a mixing time less than 54 ms.

a

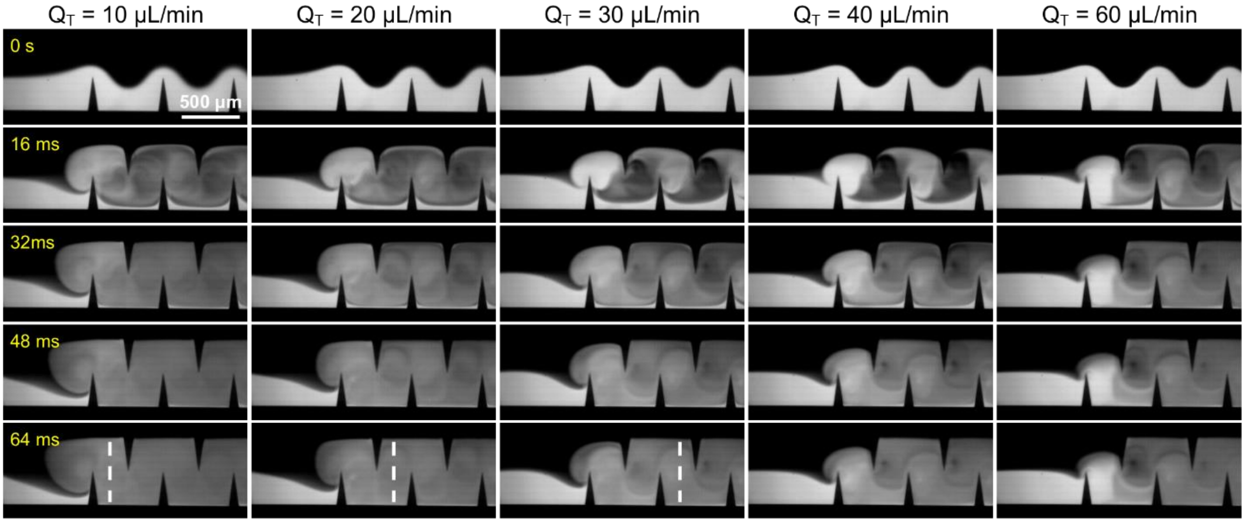

b

Q_{Total} (μL/min)	Mixing index (at dash line)	Mixing distance (μm)	Mixing time (ms)
10	0.036	< 300	< 54
20	0.040	< 600	< 54
30	0.045	< 900	< 54
40	N.A.	> 1200	> 54
60	N.A.	> 1200	> 54

Figure S11. Mixing performance presented by mixing DI water and FITC at different total flow rates of 10, 20, 30, 40, and 60 μL/min. The volumetric ratio of DI water/FITC and the driving voltage are remained at $R_{water/FITC}$ = 1 and 20 V_{PP}, respectively. (**a**) Fluorescent images showing the mixing behavior at different total flow rates. (**b**) Calculation of mixing index along the white dash line in (**a**) and the corresponding mixing time for different total flow rates. These results suggest that under a constant, low driving voltage (*i.e.*, 20 V_{PP}), the mixing performance becomes more compromised as the total flow rate is increased, thus leading to a mixing time longer than 54 ms.

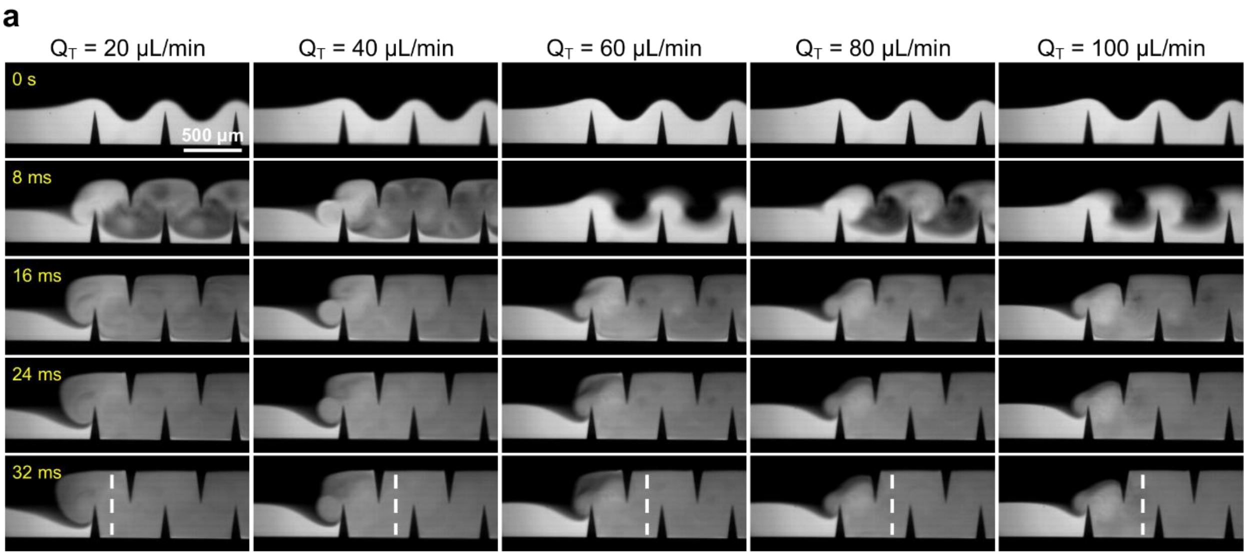

b

Q_{Total} (µL/min)	Mixing index (at dash line)	Mixing distance (µm)	Mixing time (ms)
20	0.036	< 300	< 27
40	0.040	< 300	< 13.5
60	0.045	< 600	< 18
80	0.045	< 600	< 13.5
100	0.047	< 600	< 10.8

Figure S12. Mixing performance presented by mixing the DI water and FITC at various total flow rates including 20, 40, 60, 80, and 100 µL/min, under the driving voltage of 50 V_{PP}. The volumetric ratio of DI water/FITC is remained at $R_{water/FITC}$ = 1. (**a**) Fluorescent images showing the mixing behavior at different total flow rates. (**b**) Calculation of mixing index along the white dash line in (**a**) and the corresponding mixing time for different total flow rates. These results suggest that under a constant, high driving voltage (*i.e.*, 50 V_{PP}), the mixing performance at high flow rates can be significantly improved, compared to those obtained at the same total flow rates but a low driving voltage (**Figure S11**).

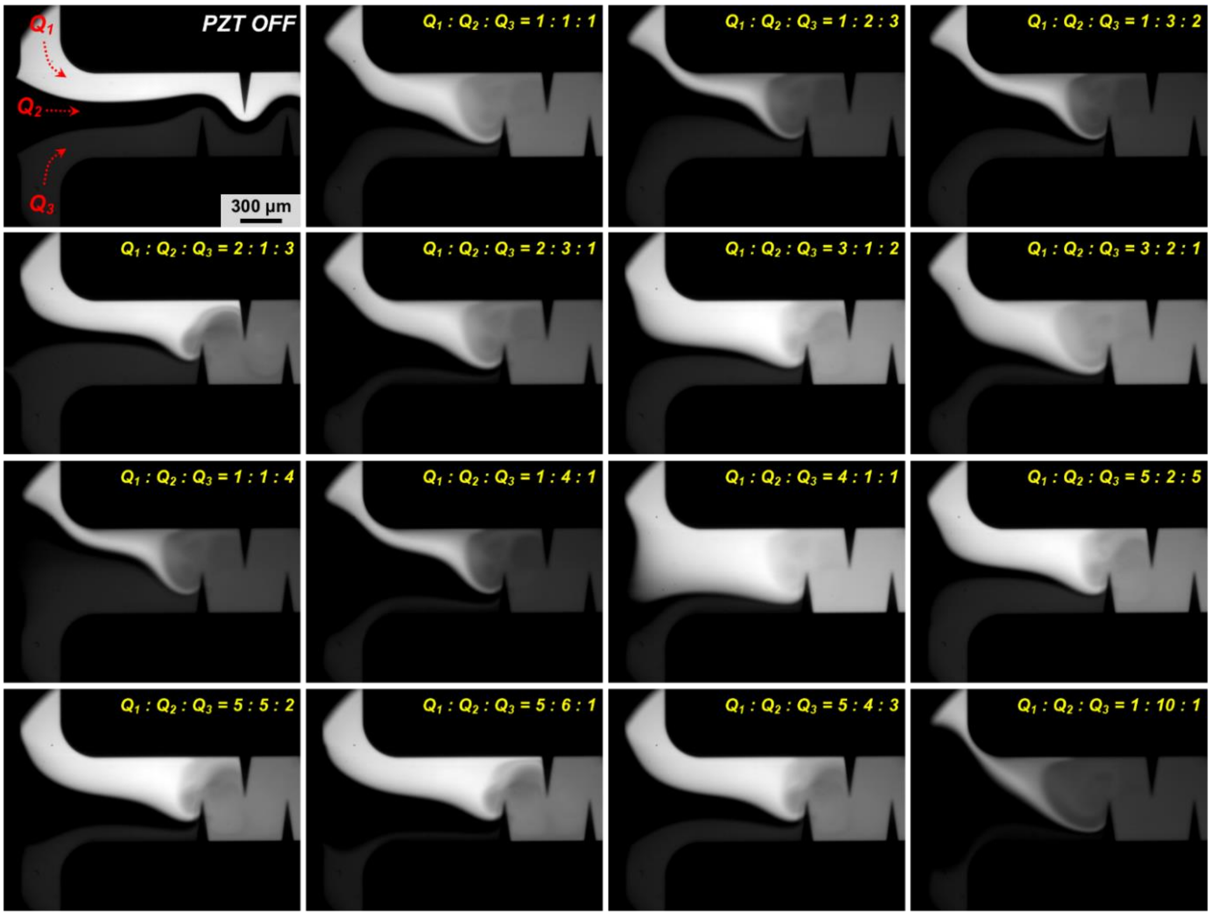

Figure S13. Experimental images showing the rapid, complete mixing of three solutions (Q_1, Q_2, and Q_3) at varying flow rate ratios using our acoustofluidic device. Regardless of flow rate ratios, the mixing of the three solutions can be achieved before they arrive at the third sharp-edge structure, which corresponds to a mixing time of ~18 ms for all the cases. These results demonstrate that our acoustofluidic device can rapidly and completely blend three liquids together at a wide range of flow rate ratios while maintaining a constant mixing time. Experiments for these results are carried out under the following conditions: a driving voltage of 30 V_{PP} and a total flow rate of 60 µL/min.

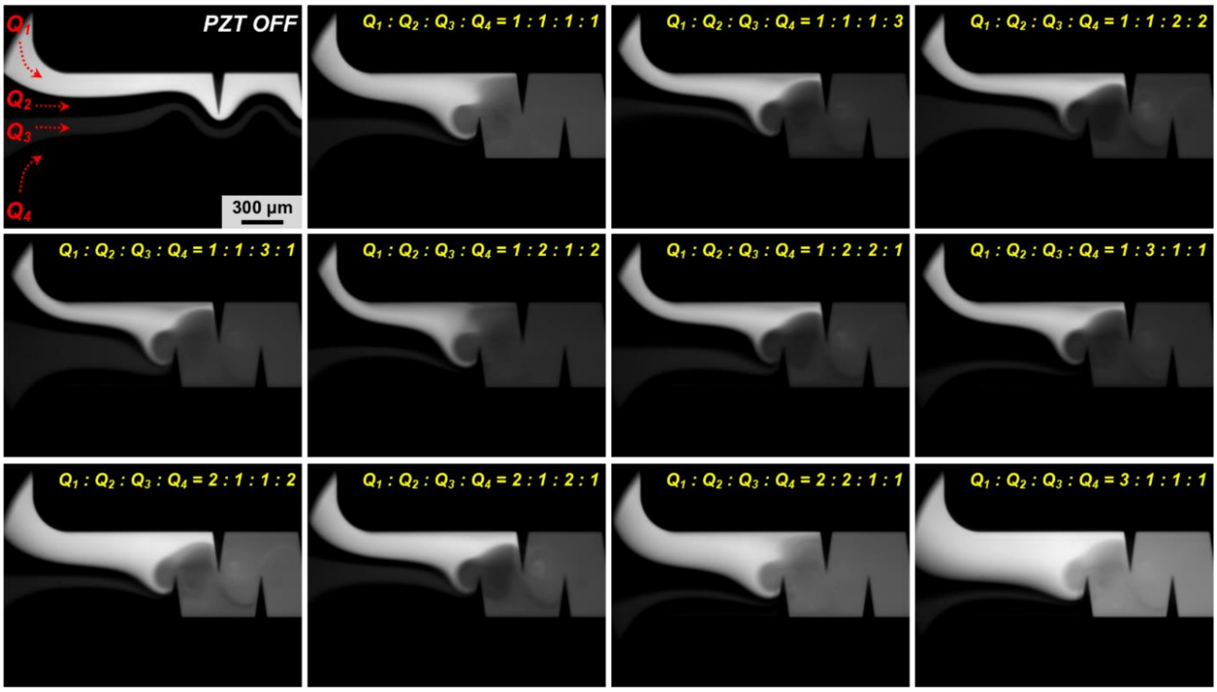

Figure S14. Experimental images showing the rapid, complete mixing of four solutions (Q_1, Q_2, Q_3, and Q_4) at varying flow rate ratios using our acoustofluidic mixing device. Likewise, regardless of flow rate ratios, the mixing of the four solutions can be achieved before they arrive at the third sharp-edge structure, which corresponds to a mixing time of ~18 ms for all the cases. These results demonstrate, once again, that our device is capable of rapidly and completely mixing multiple liquids together at a wide range of flow rate ratios while maintaining a constant mixing time. Experiments for these results are carried out under the following conditions: a driving voltage of 30 V_{PP} and a total flow rate of 60 µL/min.

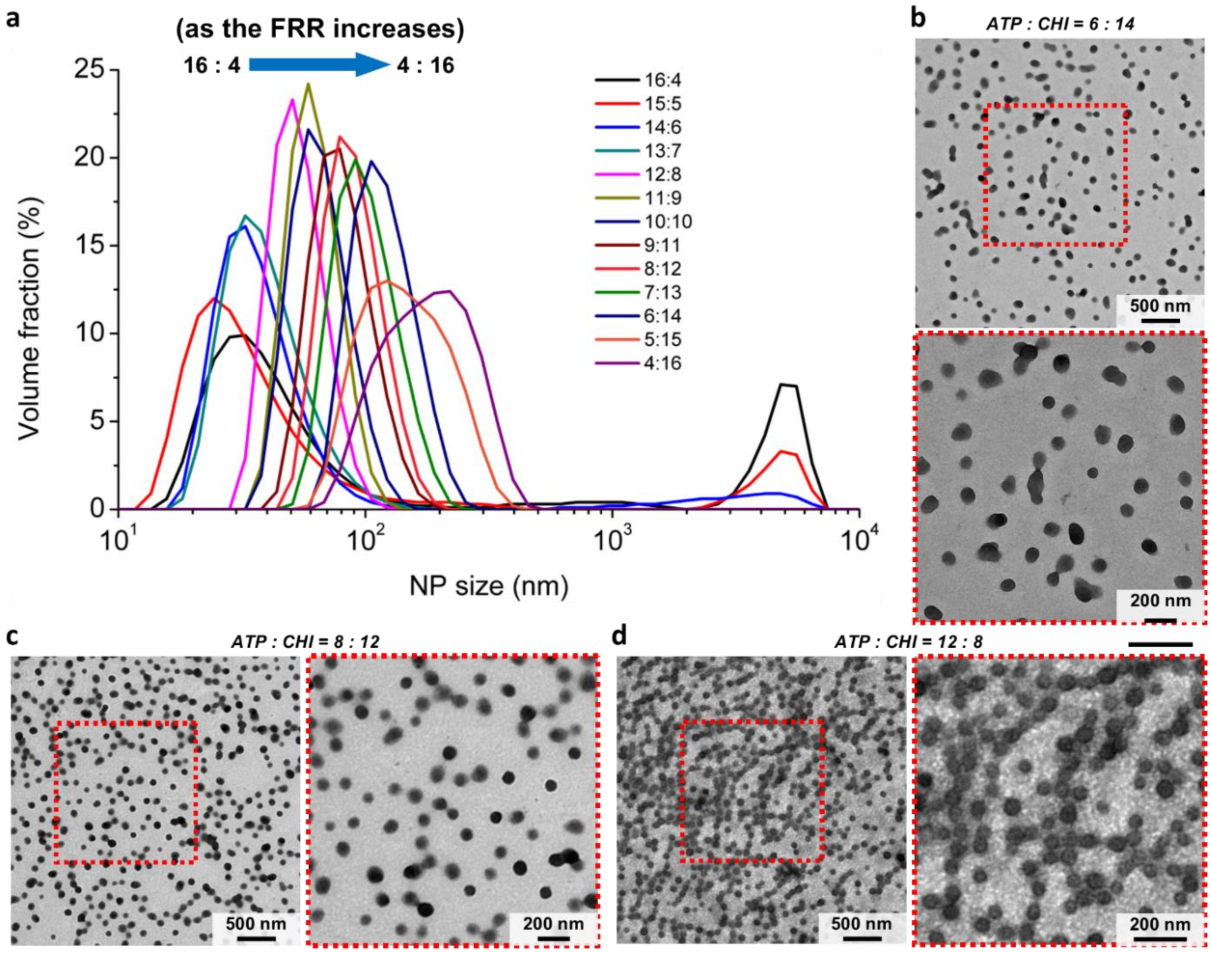

Figure S15. (**a**) Size distribution for the chitosan NPs synthesized at varying flow rate ratios. Each flow rate ratio yields a distinct size distribution, and as the flow rate ratio is reduced, the size distribution shifts gradually towards larger NPs size. (**b**)-(**d**) TEM images for the chitosan NPs synthesized at the flow rate ratio of 6:14, 8:12, and 12:8, respectively, confirming the size of chitosan NPs synthesized.

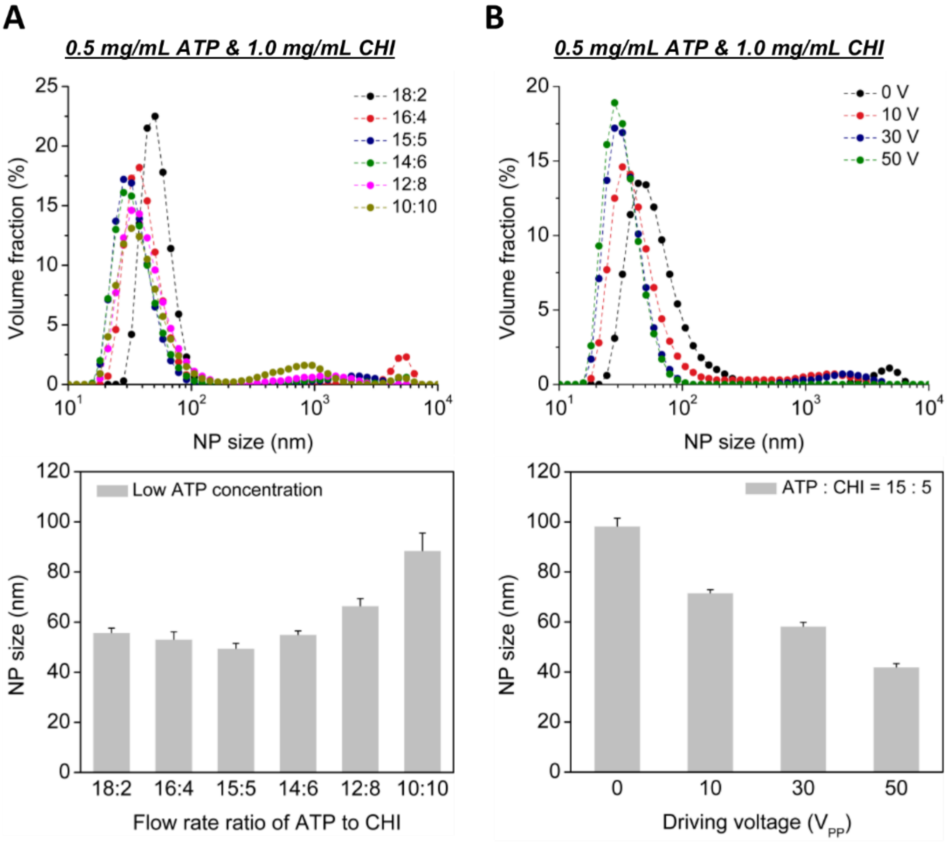

Figure S16. Synthesis of chitosan NPs at another ATP/chitosan concentration (chitosan = 1.0 mg/mL; ATP = 0.5 mg/mL). (**a**) Size distribution and average size of chitosan NPs synthesized at varying flow rate ratios. After the concentration is changed, the smallest chitosan NPs (49.5 ± 2 nm) are synthesized at the flow rate ratio of 15:5, which indicates that 15:5 is the optimal flow rate ratio at these given concentrations. (**b**) Size distribution and average size for chitosan NPs synthesized at a constant flow rate ratio of 15:5 while under different driving voltages. As the driving voltage increases, the size of chitosan NPs decreases strikingly, demonstrating our platform can control the size of chitosan NPs by controlling the mixing performance, *i.e.*, by controlling the mixing time, through adjusting the driving voltage.

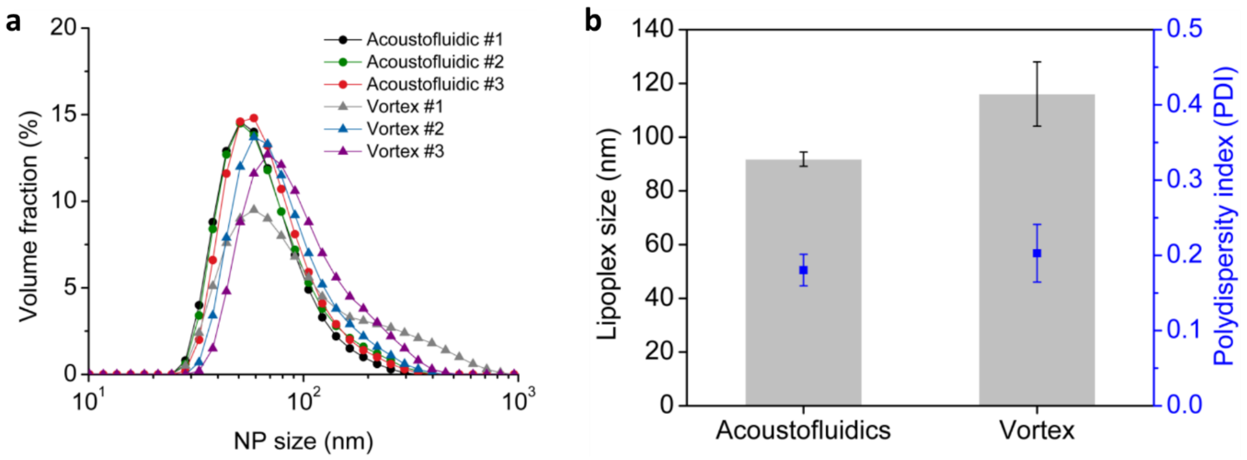

Figure S17. Synthesis of lipoplexes by acoustofluidic device and vortex mixing. (**a**) Size distribution for lipoplexes synthesized by the two approaches. Our acoustofluidic device generates size-distribution curves that nearly overlap and have higher volume fraction, while size-distribution curves from vortex mixing deviate significantly. (**b**) Average size of lipoplexes synthesized by the two mixing approaches. Compared to vortex mixing, our acoustofluidic device can synthesize lipoplexes that are smaller both in size and polydispersity index, in a reproducibly manner. These results demonstrate that the acoustofluidic device can reproducibly yield lipoplexes with tighter size distribution and smaller average size than that produced by vortex mixing. Experiments were carried out using 10 µg/mL lipofectin and 10 µg/mL pcDNA-EGFP (both dissolved in purified water), under the driving frequency of 4.0 kHz , driving voltage of 30 V_{PP}, and the flow rate of 10 µL/min for both lipofectin and pcDNA-EGFP. Error bars denote standard deviation from at least three experiments (n ≥ 3).

Table S1. Detailed average sizes for PLGA-PEG NPs synthesized using polymers of five molecular weights (MWs) at three concentrations, by vortex mixing, diffusion-based mixing (Acoustic OFF), and acoustofluidic device (Acoustic ON). Unit: nm. Data represents average ± standard deviations from three experiments (*n*=3).

5 mg/mL			
MWs \ Methods	Vortex mixing	Acoustic OFF (0 V_{PP})	*Acoustic ON (20 V_{PP})*
10K-1K	126.34 ± 10.18	186.3 ± 7.2	95.8 ± 2.0
10K-3K	268.1 ± 13.0	350.7 ± 15.0	200.6 ± 3.5
10K-5K	78.3 ± 12.1	105.7 ± 2.8	73.1 ± 1.58
20K-5K	76.2 ± 9.51	109.8 ± 2.9	73.3 ± 1.66
40K-5K	82.51 ± 8.32	112.3 ± 1.05	81.1 ± 1.83

10 mg/mL			
MWs \ Methods	Vortex mixing	Acoustic OFF (0 V_{PP})	*Acoustic ON (20 V_{PP})*
10K-1K	161.87 ± 21.18	256.2 ± 8.93	137.0 ± 1.50
10K-3K	338.45 ± 17.00	431.3 ± 23.0	306.8 ± 4.51
10K-5K	108.9 ± 15.00	143.4 ± 4.10	83.2 ± 2.43
20K-5K	99.4 ± 7.730	125.7 ± 8.96	79.85 ± 1.33
40K-5K	87.23 ± 10.93	121.2 ± 0.75	90.03 ± 1.71

20 mg/mL			
MWs \ Methods	Vortex mixing	Acoustic OFF (0 V_{PP})	*Acoustic ON (20 V_{PP})*
10K-1K	449.6 ± 78.8	608 ± 6.5	249.53 ± 10.54
10K-3K	626.5 ± 81.5	912.3 ± 10.51	360.37 ± 6.38
10K-5K	132.5 ± 25.1	205.3 ± 7.43	132.6 ± 1.73
20K-5K	118.0 ± 17.6	231.1 ± 6.67	131.6 ± 3.7
40K-5K	111.76 ± 15.5	246.3 ± 5.71	138.3 ± 0.49

Table S2. Detailed flow rates used in the synthesis of TTF- $HAuCl_4$ nanohybrids.

**Flow rate ratio (TTF to $HAuCl_4$)**	**0.086**	**0.428**	**1.43**	**4.28**	**8.56**
TTF	1.6 μL/min	6 μL/min	11.8 μL/min	16.2 μL/min	17.9 μL/min
$HAuCl_4$	18.4 μL/min	14 μL/min	8.2 μL/min	3.8 μL/min	2.1 μL/min
